# Incorporation of uncertainty to improve projections of tidal wetland elevation and carbon accumulation with sea-level rise

**DOI:** 10.1371/journal.pone.0256707

**Published:** 2021-10-20

**Authors:** Kevin J. Buffington, Christopher N. Janousek, Bruce D. Dugger, John C. Callaway, Lisa M. Schile-Beers, Evyan Borgnis Sloane, Karen M. Thorne

**Affiliations:** 1 U.S. Geological Survey, Western Ecological Research Center, Davis, California, United States of America; 2 Department of Fisheries, Wildlife, and Conservation Sciences, Oregon State University, Corvallis, Oregon, United States of America; 3 University of San Francisco, San Francisco, California, United States of America; 4 Silvestrum Climate Associates, San Francisco, California, United States of America; 5 California Coastal Conservancy, Oakland, California, United States of America; Universidade de Aveiro, PORTUGAL

## Abstract

Understanding the rates and patterns of tidal wetland elevation changes relative to sea-level is essential for understanding the extent of potential wetland loss over the coming years. Using an enhanced and more flexible modeling framework of an ecosystem model (WARMER-2), we explored sea-level rise (SLR) impacts on wetland elevations and carbon sequestration rates through 2100 by considering plant community transitions, salinity effects on productivity, and changes in sediment availability. We incorporated local experimental results for plant productivity relative to inundation and salinity into a species transition model, as well as site-level estimates of organic matter decomposition. The revised modeling framework includes an improved calibration scheme that more accurately reconstructs soil profiles and incorporates parameter uncertainty through Monte Carlo simulations. Using WARMER-2, we evaluated elevation change in three tidal wetlands in the San Francisco Bay Estuary, CA, USA along an estuarine tidal and salinity gradient with varying scenarios of SLR, salinization, and changes in sediment availability. We also tested the sensitivity of marsh elevation and carbon accumulation rates to different plant productivity functions. Wetland elevation at all three sites was sensitive to changes in sediment availability, but sites with greater initial elevations or space for upland transgression persisted longer under higher SLR rates than sites at lower elevations. Using a multi-species wetland vegetation transition model for organic matter contribution to accretion, WARMER-2 projected increased elevations relative to sea levels (resilience) and higher rates of carbon accumulation when compared with projections assuming no future change in vegetation with SLR. A threshold analysis revealed that all three wetland sites were likely to eventually transition to an unvegetated state with SLR rates above 7 mm/yr. Our results show the utility in incorporating additional estuary-specific parameters to bolster confidence in model projections. The new WARMER-2 modeling framework is widely applicable to other tidal wetland ecosystems and can assist in teasing apart important drivers of wetland elevation change under SLR.

## Introduction

Tidal wetlands are some of the most productive ecosystems in the world and provide habitat to migratory birds, fish, and terrestrial wildlife [1]. Due to coastal development, diking, and draining, tidal wetlands have been lost globally [2], which has contributed to a decline in biodiversity and many wildlife species [3, 4]. These ecosystems help maintain good water quality, reduce turbidity, and attenuate storm and flood waters [5]. Tidal wetlands have also been shown to sequester and store carbon in their organic soils over hundreds of years [6]. Saltwater and brackish tidal wetlands are dynamic and evolving coastal ecosystems, but they are vulnerable to a variety of anthropogenic stressors including human development, eutrophication, freshwater availability, and climate change.

An important climate change impact to the coastal zone is accelerating sea-level rise (SLR), which can lead to wetland submergence when rates of SLR exceed vertical accretion [7–10]. For centuries, coastal wetland elevation has largely been in balance with relative rates of SLR due to accretion or transgression processes [11]. However, due to greenhouse gas emissions and atmospheric warming, global SLR rates are accelerating [12], with regional hotspots that exceed the global mean of 3.1 mm yr^-1^ [13, 14]. For the Pacific coast of North America, relative SLR projections for 2100 range from less than 40 cm to over 200 cm, with the magnitude dependent on realized greenhouse gas emissions over the coming century [15]. Shorter-term meteorological and oceanographic phenomena such as storm surges, atmospheric rivers, and El Niño-Southern Oscillation events are also anticipated to add to the coastal flooding impacts of long-term SLR itself [16, 17]. Coastal inundation from SLR and storms could impact and displace millions of people with severe impacts to coastal economies [17]; however tidal wetlands can mitigate some of these impacts by providing flood protection benefits [e.g., 18–20]. Understanding tidal wetland persistence under SLR is crucial for coastal adaptation planning, resource management, and the development of mitigation efforts.

The ability of tidal wetlands to build elevation relative to local SLR depends on the strength of three major processes. First, mineral sediment deposition is a key component of vertical growth of the wetland surface. Sediment inputs are a function of sediment availability in the estuary, the duration of tidal inundation, and trapping efficiency of tidal wetland vegetation [21, 22]. Second, vertical growth of wetlands is aided by organic matter production, mainly via belowground productivity by emergent vegetation that builds peat soils [23, 24]. Lastly, organic matter decomposition and sediment compaction can reduce soil profiles and offset elevation gains. Biogeomorphic conditions such as hypersalinity, soil saturation, or eutrophication can lead to lower plant productivity, which can also limit belowground organic matter contributions to soil volume [25–28].

Long-term wetland persistence is also affected by the rate of seaward edge erosion and the availability of accommodation space in adjacent upland areas into which wetlands can migrate upslope with SLR [29]. However, if upland areas are inaccessible due to coastal development or barriers, vertical accretion is key to prevent loss. Most estuaries have been modified by human development with alterations to the landscape that affect one or more of these accretion or migration process [30]. For instance, along the Pacific coast of the United States, many estuaries are located either adjacent to intensely developed urbanized areas or in coastal watersheds with steep topography that limit their potential to migrate inland [31]. In other regions, such as along the U.S. Atlantic coast, wetland migration may be possible [29].

Watershed modifications by anthropogenic activities (e.g., dams, dredging, diking, land use, agriculture) can result in novel conditions that change mineral sediment availability for vegetated tidal wetlands [32]. In the Lower Mississippi River, Louisiana the sediment load has decreased by 80% since 1850 due to levee construction and water diversion [33], while sedimentation rates in Chesapeake Bay, Virginia have increased since European settlement [34]. The San Francisco Bay Estuary in California had a suspended sediment load and contaminant increase due to hydraulic mining between 1852–1884 during the California Gold Rush [35], which has since decreased. Tidal wetland accretion responses to these altered sediment landscapes is key in understanding SLR vulnerability over the next century.

Modeling has successfully been used to assess wetland vulnerability to SLR with evolving sophistication relative to earlier work that often lacked site-specific information or mechanistic feedbacks [36]. More robust modeling approaches for projecting long-term change in wetlands such as the Marsh Equilibrium Model (MEM; [9, 37, 38]) and the Wetland Accretion Rate Model of Ecosystem Resilience (WARMER; [39, 40]) incorporate the major functional processes that affect marsh elevations: sedimentation rates, SLR, plant productivity, compaction, and decomposition. The relative contributions of these processes to accretion and elevation change will differ among wetland types due to unique combinations of geomorphic setting, climate, plant community composition, tidal range, and sediment availability. For instance, plant productivity along elevation gradients can differ by plant species [40, 41], and the contributions of organic materials and mineral sediments to accretion can vary within and across estuaries and climate zones [42]. Wetland elevation models are often developed and parameterized for specific geographic regions or well-studied plant species (usually *Spartina alterniflora* in USA), and the transferability of such parameters to other wetlands is unknown. Many modeling efforts also do not incorporate plant community change over time, even though it is well documented that tidal wetland vegetation composition can vary markedly with elevation, inundation, and salinity [43, 44], and that climate change is projected to alter both flooding and salinity gradients in estuaries [45, 46]. Simulations of wetland carbon storage range from relatively simple accounting approaches [47] to more complex biochemical models [48]. Finally, SLR wetland models typically do not incorporate parameter uncertainty, which leads to unknown probability distributions of wetland elevations under different SLR scenarios. To increase confidence in model projections, data on vegetation type and transitions, salinity responses, and sediment availability that are specific to geographic regions under consideration should be incorporated.

In this study we developed and implemented a revised wetland elevation model [39] that addresses several previous model limitations. Our approach adds new functionality and is transferable across coastlines. Specifically, we expanded the model to incorporate, in addition to SLR scenarios: (1) plant assemblage transitions, (2) salinity effects on productivity, (3) changes in sediment availability, (4) accounting of blue carbon accumulation rates, and (5) Monte Carlo functionality to quantify uncertainty. We used the model to evaluate the synergistic effects of changes in salinity, vegetation type, sediment supply, and SLR on wetland elevations, and evaluate the SLR rate threshold for marsh persistence. Next, we evaluated the sensitivity of model outcomes to variability in key parameter inputs including sediment supply, SLR, organic matter decomposition, and species-specific relationships between elevation and plant productivity. Finally, we examined how a key ecosystem service–carbon sequestration–is projected to change across a salinity gradient in the San Francisco Bay Estuary (SFBE) under different SLR, sediment, and salinity scenarios.

## Materials and methods

### Study region

SFBE is a shallow estuary located in north-central California, USA, that experiences a Mediterranean climate with mild, wet winters and hot, dry summers. The SFBE ecosystem has undergone a dramatic transformation since European settlement in the mid-1800s, with an 80% reduction in the area of tidal wetlands, due to diking, filling, or conversion to agriculture [49]. Current bayland (marsh and mudflat) extent is about 32,000 ha [50], with over 40,000 ha targeted for restoration [51]. The wetlands that remain provide critical habitat to fish and wildlife, including many federal and/or state threatened and endangered species such as the Ridgeway’s rail (*Rallus longirostris obsoletus*), California black rail (*Laterallus jamaicensis coturniculus*), and the salt marsh harvest mouse (*Reithrodontomys raviventris*); the estuary is also an important stopover for migratory birds along the Pacific flyway [1]. Accretion in Pacific coast tidal saline and brackish marshes is generally dominated by mineral deposition, evidenced by high mineral accumulation rates, high soil bulk densities, and relatively low soil organic matter content [52].

**Fig 1:**
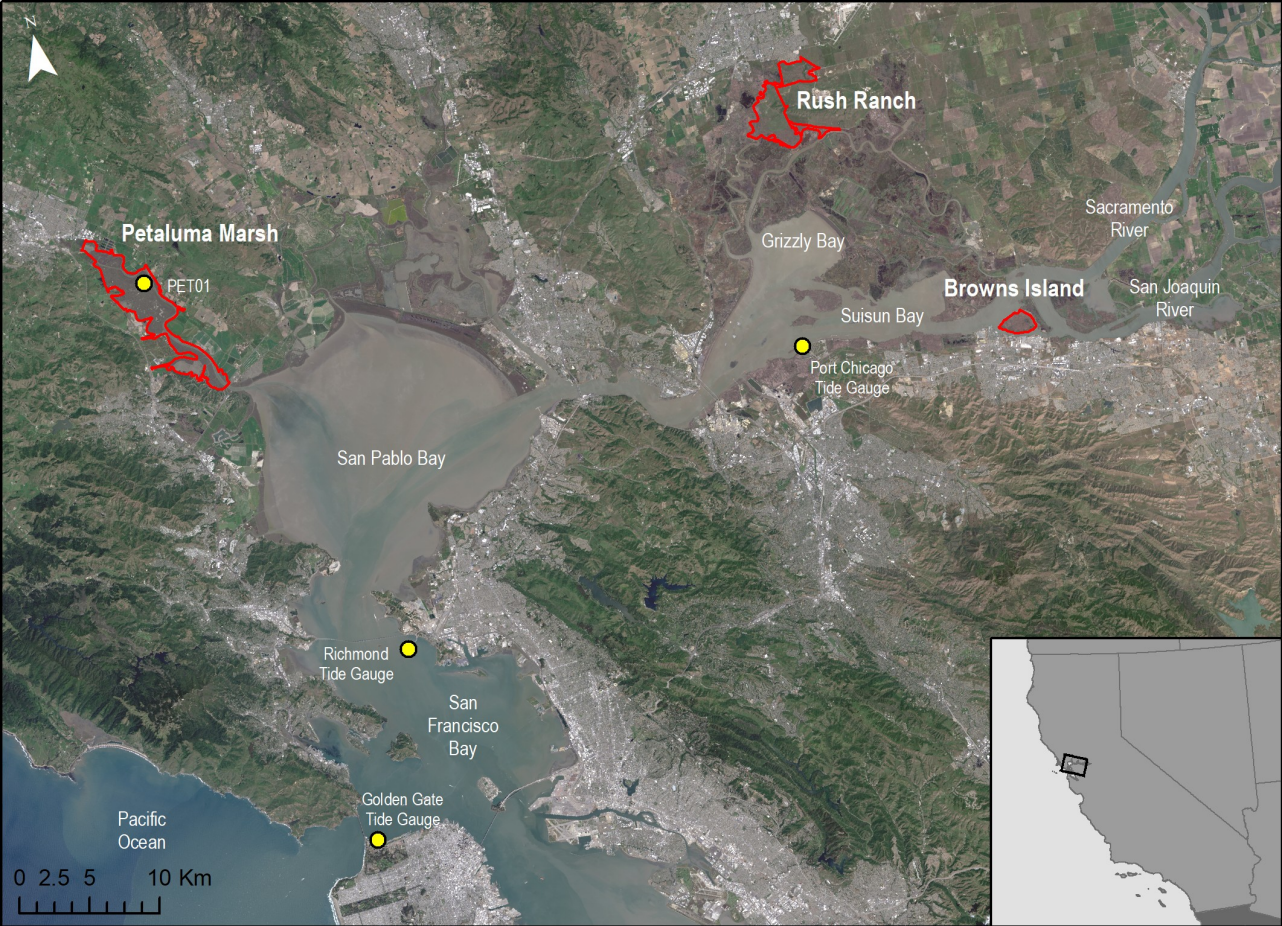
Study site map. Location of study sites and tide gauges within the San Francisco Bay estuary.

We conducted modeling for three tidal wetland sites in the SFBE that occur along a gradient of tidal inundation, water salinity, and climate (Fig 1). Our most saline site, Petaluma Marsh Wildlife Area (Petaluma, 38.191°N, 122.55°W; permission granted by California Department of Fish and Wildlife), is a valley marsh [53] located north of San Pablo Bay in the Petaluma River Valley and is one of the largest remaining and least-disturbed tidal marshes in San Francisco Bay. Rush Ranch Open Space (Rush Ranch, 38.193°N, -122.022°W; permission granted by San Francisco Bay National Estuarine Research Reserve) is a large remnant of the extensive brackish marshes that historically occurred throughout Suisun Bay. Browns Island Regional Preserve managed by East Bay Regional Parks District (Browns Island, 38.039°N, 121.866°W; permission granted by East Bay Regional Parks, permit 821) is an oligohaline island marsh near the confluence of the Sacramento and San Joaquin rivers at the western end of the Delta. All sites in the study have an unrestricted tidal range and experience a mixed, semi-diurnal tide.

### WARMER-2 model description

We modified WARMER [39], a 0-D soil cohort model for tidal wetlands, to compare several organic productivity functions and incorporate a productivity response to changing salinity, while also propagating parameter uncertainty into projections of wetland elevation with accelerating SLR. This adapted modeling framework, WARMER-2, incorporates the dominant above- and belowground processes that control elevation relative to mean sea level that can be summarized with the general equation:

$$E_{t+1}=E_{t}+MAR_{E(t)}+OAR_{E(t)}-DECOMP_{t}-SLR_{t}$$ (1)

where *E* is marsh elevation relative to mean sea level, *MAR*_*E*_ is annual rate of mineral deposition at elevation *E*, *OAR*_*E*_ is total organic matter production at elevation *E*, *DECOMP*_*t*_ is the decomposition rate, and *SLR*_*t*_ is the annual amount of SLR. Empirical data, typically from dated soil cores, are used to calibrate the mineral (MAR) and organic accumulation rate (OAR) functions in WARMER-2.

We used sediment accumulation rates from replicate cores collected at Petaluma, Rush Ranch, and Browns Island dated using ^210^Pb profiles [52] to calibrate the mineral and organic deposition functions in WARMER-2. Depth profiles of bulk density and percent organic matter, calculated in 2 cm increments, provided estimates of MAR and OAR:

$$OAR=\sum_{a}^{0}o\rho_{s}$$ (2)

$$MAR=\sum_{a}^{0}(1-o)\rho_{s}$$ (3)

where *a* is depth (cm), based on 50 years of vertical accretion from ^210^Pb dating, *ρ*_s_ is bulk density, and *o* is percent organic matter from loss-on-ignition.

In WARMER, mineral deposition is a function of inundation frequency and elevation, calibrated with the annual MAR of soil cores at a known elevation [39]. This approach produces reasonable predictions of mineral deposition across much of the vegetated marsh, capturing the expected negative relationship between elevation and deposition. At lower elevations, where vegetated marsh transitions to unvegetated mudflat, this approach leads to unrealistically high rates of deposition since it assumes erosion does not occur. To better account for the dynamics in this critical zone, we adopted a point-based sediment deposition model based on prior work [54–57]. This model balances sediment deposition flux, *Q*, as the result of settling, *Q*_*ds*_, and erosion, *Q*_*e*_, caused by tidal current shear stress,

$$\frac{dQ}{dt}=Q_{ds}-Q_{e}$$ (4)

Shear stress, *τ*_0_, is defined by

$$\tau_{0}=\lambda_{\gamma }U$$ (5)

where *γ* is the specific density of water (9.807 kN m^-3^), *U* is the horizontal water velocity in m s^-1^, defined as

$$U=n\frac{D}{\lambda }$$ (6)

where *n* is the instantaneous change in the simulated water level timeseries, *D* is water depth (m), and *λ* is a bottom friction coefficient defined as

$$\lambda =\frac{8}{3\pi }\;\frac{U_{0}}{K^{2}}$$ (7)

where *U*_*0*_ is the maximum tidal current (assumed to be 0.2 m s^-1^ [57]), and *K* is Chezy’s friction coefficient, assumed to be 10 m^1/2^ s^-1^ [57].

Deposition caused by settling, *Q*_*ds*_ is defined as,

$$Q_{ds}=\left\{ \begin{array}{ll} w_{s}\;C\;(1-\frac{\tau_{0}}{\tau_{d}}) & if\;\tau_{0}<\tau_{d}\\ 0 & if\;\tau_{0}\geq \tau_{d} \end{array}\right.$$ (8)

where *w*_*s*_ is the settling velocity (m s^-1^, assumed to be a constant 1.0x10^-4^ and calibrated using sediment deposition data from reference [22]), *C* is the depth-averaged suspended sediment concentration, and *τ*_d_ is the shear stress limit above which sediment flocs do not settle and remain in the water column (0.1 N m^-2^). Erosion flux, Q_e_ is defined as,

$$Q_{e}=\left\{ \begin{array}{ll} Q_{e0}\;(\frac{\tau_{0}}{\tau_{e}}-1) & if\;\tau_{0}>\tau_{e}\\ 0 & if\;\tau_{0}\leq \tau_{e} \end{array}\right.$$ (9)

where *τ*_e_ is the critical shear stress needed to break up the bed (0.4 N m^-2^), *Q*_*e*0_ is an empirical coefficient = 1/ρ_s_ x 3.0x10^-4^ m s^-1^, with ρ_s_ = 2600 kg m^-3^. Suspended sediment concentrations (SSC) were assumed constant during flood tides, but on ebb tide the instantaneous sediment concentration is reduced as particles settle on the surface,

$$\frac{dDC}{dt}=-w_{s}C+C\frac{dh}{dt}$$ (10)

where *h(t)* is the water level (m, MSL) and *D(t)* is instantaneous water depth (m; *D(t)-z*).

#### Root growth

Organic matter from roots, *r*_*g*_, was distributed non-linearly with depth, according to the function:

$$r_{g}=e^{-r_{2}(V+d)Vr_{2}}$$ (11)

where *r*_*2*_ is the decay coefficient, *V* is the volume of the annual soil cohort (cm^3^) and *d* is the depth of the cohort within the soil column. Living roots are also distributed according to *r*_*g*_, with a constant density of 0.2 (maximum value from [58]).

#### Decomposition

Organic matter is divided into refractory and labile pools based on *r*, the relative proportion of organic matter in the bottom 4 cm of the soil core compared with the top 4 cm.


$$r=\frac{om_{bottom}}{om_{top}}$$ (12)

Average *r* values were calculated across each site. Decomposition was assumed to occur at different rates depending on the age class (*i*; 1, 2, or 3+ years old) and depth (*d*) of labile organic material,

$$F_{Mo}(i,d)=1-A(i)e^{-k_{decomp}d)}$$ (13)

where *A* is a coefficient for age class. The influence of depth on decomposition (k_decomp_) was set to a constant 0.05; this is a simplification compared to WARMER and reflects the lack of data on how decomposition rates change with soil depth.

#### Soil volume

We made a substantial change in how soil volume was calculated in WARMER-2. In WARMER, density constants for mineral and organic matter were employed to calculate volume; however, these general constants may not be representative of local conditions. Instead, bulk density of each cohort was calculated from an ideal mixing model for organic and mineral sediments [59], where:

$$\rho_{s}=\frac{1}{\frac{o}{k_{1}}+\frac{1-o}{k_{2}}}$$ (14)

We calibrated the mixing model using the interval soil core data and estimated *k*_*1*_ and *k*_*2*_ as 0.0823 and 1.876, respectively. Compaction and porosity are no longer explicitly calculated as in WARMER; rather, down-core organic decomposition leads to a greater mineral fraction and higher bulk density that reduces cohort volume and causes compaction.

#### Species transition model

As wetland elevation changes in response to SLR, plant assemblages are likely to shift because many species tend to show vertical zonation related to inundation [44, 60]. Accounting for transitions among dominant species is important for a more accurate estimate of organic matter accumulation, sediment accretion, and wetland elevation. We used a dynamic population model to simulate changes in plant percent cover and corresponding organic matter accumulation,

$$\frac{dP}{dt}=P_{i}r1_{i}[1-\frac{p_{i}}{{\left(1-P_{i}\right)}_{S(z,i)}}]$$ (15)

where *P* is the percent cover for species *i*, *P*_*j*_ is the total fractional cover excluding species *i*, *r1*_*i*_ is the maximum growth rate for species *i*, and *S(z*,*i)* is an elevation probability distribution function [0–1] for species *i* that was obtained from field surveys. Maximum growth rates were constrained through calibration and by comparing time-series measurements of plant height in marsh organ experiments conducted in the estuary [41, 61, 62]. Competition was not included as we assumed that the probability density function of a species’ occurrence in the field included both physiological limitations and biotic interactions. Soil organic matter accumulation (SOM) was defined as a function of elevation and plant percent cover,

$$SOM_{t}=\sum P_{i}O_{i}(z)$$ (16)

where *O*_*i*_*(z)* is the species-specific organic production curve, calibrated to the site-specific OAR from the soil cores [52].

*O* is a function of inundation time and species-specific productivity. The original WARMER model implemented a unimodal function based on field experiments with *S*. *alterniflora* [37], a species common to Atlantic and Gulf coast salt marshes in the United States. However, to provide regionally-relevant links between inundation and productivity, we developed functional relationships between wetland elevation and productivity using data for five common tidal wetland species from the SFBE. Inundation-relationships for all species were developed from marsh organ experiments (sensu [63]). *Spartina foliosa*, *Bolboschoenus maritimus*, and *Salicornia pacifica* are common in the more saline regions of SFBE and each was shown to have non-linear relationships with inundation, ranging from unimodal responses in *S*. *foliosa* and *B*. *maritima* to a non-linear monotonic decline in *S*. *pacifica* [41]. Combined data from two marsh organ experiments at Rush Ranch that used similar methods, but different elevations, provided data on the biomass response of the common brackish marsh species, *Schoenoplectus americanus* to inundation [61, 62]. Finally, data for *Schoenoplectus acutus*, which occurs from brackish to freshwater tidal marshes, was available from [61]. We used second order least squares polynomial regression to define the functional shape of the organic production relationship with elevation for all five species, predicting total dry biomass with percent time inundated.

We calculated annual carbon accumulation rates (CAR) by assuming that soil organic matter consisted of 42% carbon (SE = 0.47%, n = 16), which was the average C accumulation proportion across Petaluma, Rush Ranch, and Browns Island soil cores [52].

### Calibration

To characterize physical and biological differences in accretion and plant communities among the sites, we used a combination of published data sources, modeled data, and field sampling. We determined the distribution of dominant vegetation species and elevation distribution of species using a real-time kinematic GPS (Leica GS15) at each site using field survey data collected in 2016 along transects. At even intervals along the transects, we assessed plant species presence/absence and percent cover in 0.25 m^2^ plots. From the presence/absence plant data, we computed frequency of occurrence and the median elevation of species occurrence at each site.

We determined tidal range at the sites by using recent NOAA tide gauge data (station 9415423 at Lakeville, CA for Petaluma marsh) or by determining tidal datums from subtidal water level loggers (Solinst, Georgetown, ON) that we installed in stilling wells in deep tidal channels at each site (Rush Ranch, Browns Island). We collected water level data every six minutes for 10 to 18 months per site, corrected the raw pressure data for changes in barometric pressure and density differences, and computed tidal datums (MHHW, MTL, MLLW) following established methods [64].

**Fig 2:**
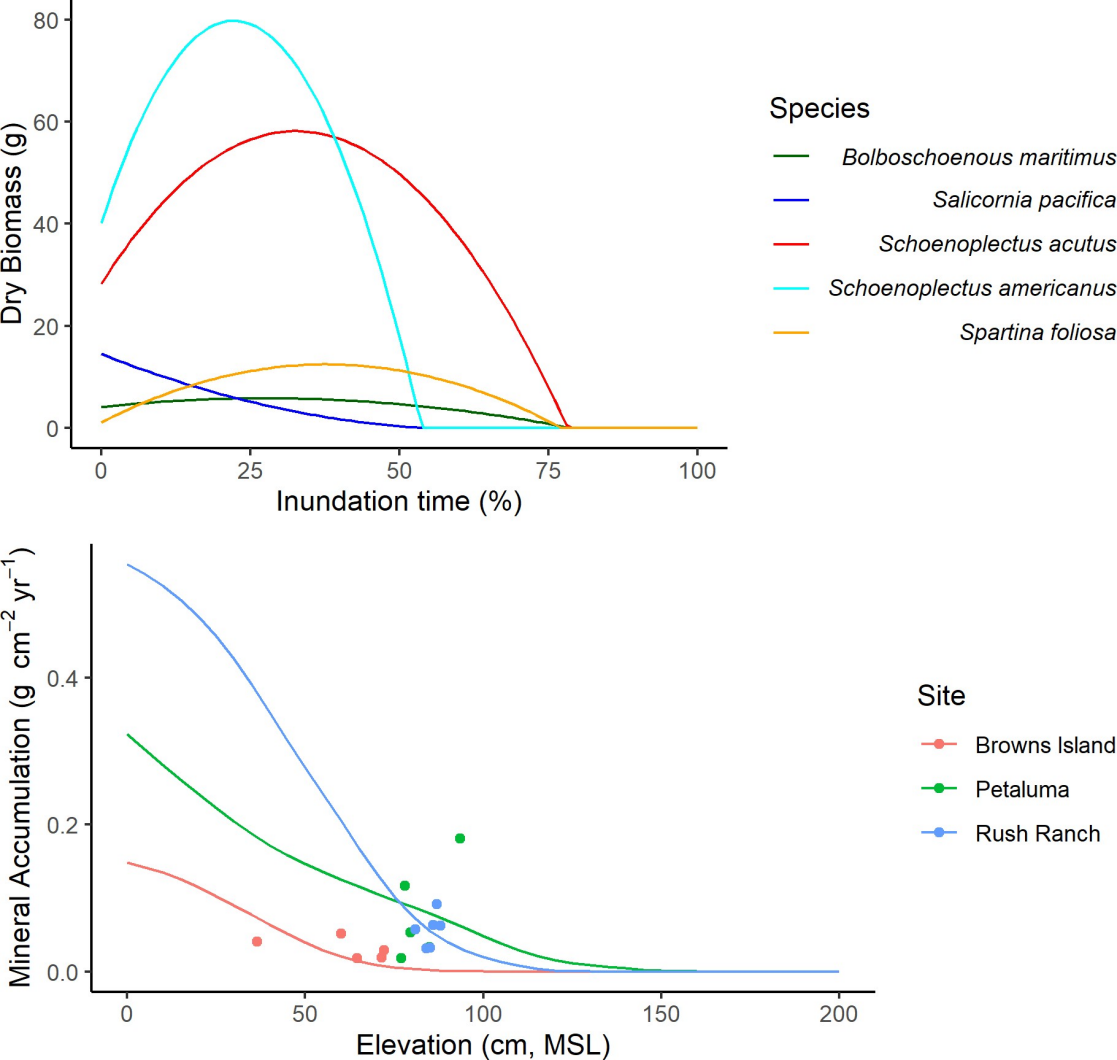
Mineral and organic accumulation functions. Organic productivity curves by percent annual inundation time for each species (top). Mineral accumulation curves, calibrated to the soil core accumulation rates at each site (bottom).

One year of water level elevations (15-minute) were derived from nearby NOAA gauge tidal harmonic constituents (Petaluma: Richmond gauge #9414863; Rush Ranch and Browns Island: Port Chicago gauge #9415144). The amplitude component of each set of harmonic constituents was scaled to the site-specific tide range (Table 1). The mineral deposition model was run for one year at C = 1 mg L^-1^ across a range of elevations; the resulting predictions of deposition were then calibrated to the soil core MAR (Fig 2).

**Table 1:** Site characteristics and model parameters (mean, standard deviation [SD]).

Parameter	Petaluma	Rush Ranch	Browns
Mean sea level (m, relative to NAVD88)	1.02	1.12	1.095
Tide range (m; MHHW-MLLW)	1.92	1.76	1.39
Mineral calibration (SSC, mg/L)	69.0 (60.8)	75.3 (40.3)	19.0 (10.1)
Root:Shoot	0.05	0.05	0.05
Annual decomposition (k; yr 1, 2, 3)	0.69, 0.91, 0.94	0.55, 0.83, 0.89	0.55, 0.83, 0.89
Annual decomposition SD	0.05	0.05	0.05
Refractory organic matter (%)	66.2 (17.8)	50.5 (27.3)	39.4 (26.0)

During MAR calibration, we accounted for the core-specific vertical accretion rate and observed changes in sea level over the last 100 years. We fit a second-order polynomial to the annual mean sea level from the NOAA Golden Gate tide gauge (station #9414290, 1900–2019; 0.1976t + 2.14e-4t^2^). The 100-year old surface elevation (relative to MSL) of each core was determined and used as the initial elevation in the calibration function (zMSL_100yrs_ = zNAVD88_present_− 100*accretionRate–[MSLpresent−SLR_100yrs_]). The calibration function then tracked annual changes in both accretion and MSL over 100 years; calibration resulted in an average MAR representing the depositional environment over the last 100 years, and because the calibration coefficient for this simple deposition model scales linearly with *C*, it provides an estimate of mean suspended sediment across the soil core locations. The long-term accumulation rate from soil cores incorporates the various processes that can affect mineral deposition, including capture by plants and distance from sediment source.

Core-derived OAR are likely closer to net organic deposition rather than gross deposition (especially in older, deeper layers); however, estimates of gross deposition are required in the model since decomposition is explicitly calculated. To address this, we included an additional calibration step to calculate a scaling factor to transform organic deposition to a gross organic deposition rate (S2 Table in S1 File). We iteratively ran WARMER-2 across each calibration core with average *C*_*0*_ and different organic deposition scalers, seeking to minimize the difference between core-derived organic accumulation and the modeled average organic deposition rate over the last 50 years of a 100-year simulation. As with mineral calibration, we used the core-derived accretion rate to estimate the 100-year depth of the soil core and adjusted the initial elevation of each calibration core to account for both the 100-year depth and total change in mean-sea level (21.9 cm).

### Implementation

We ran WARMER-2 across initial wetland elevations ranging from -0.50 to 2.0 m (MSL) in 0.1 m intervals, and from 2.5 to 5 m in 0.50 m intervals. The wide range of initial elevations was needed to accommodate the potential for upland transgression at Rush Ranch and Petaluma. For each combination of initial elevation and organic matter function, a 200-year spin-up was run to generate an initial soil profile and a constant rate of sea-level rise was determined that resulted in a final elevation equal to the initial elevation. Model projections for each year were then interpolated across a DEM that represented conditions in 2015. Lidar-derived DEMs for each site were corrected for vertical bias caused by vegetation using the LEAN method [65]. We used existing corrections for Petaluma [66] and Rush Ranch [67], and we developed a new correction for Browns Island (original RMSE = 0.474 m, LEAN RMSE = 0.178 m).

### Validation

Validation of future projections of marsh elevation under accelerating SLR is not possible, and there are few long-term monitoring datasets available to make independent assessments. However, we did use several approaches to test the validity of the WARMER-2 model. First, we compared the spin-up SLR rate for mean ± SD marsh elevation to the long-term average SLR rate. Second, we compared the modeled calibration profiles of percent organic matter and bulk density with data from soil cores. Finally, we compared average recent accretion rates from Surface Elevation Tables at each site (n = 4 per site; Thorne et al. *in review*) against the mean modeled accretion rate from short (5-year) model runs as an independent validation of the model. The observed annual deviations in MSL at the Richmond and Port Chicago NOAA gages were used to inform changes in sea level (2016–2020) relative to site-specific MSL.

### Scenarios

#### Sediment availability

Hydraulic mining during the gold rush of the late 1800s produced a pulse of sediment that was delivered to San Francisco Bay until ~1999 [32]. Accretion rates estimated using soil core dating are based mostly on those historic sediment supply conditions and thus may not be an accurate analog for future conditions. Additionally, future climate change projections for precipitation and freshwater discharge are highly variable and will affect the availability of suspended sediment for deposition. Given the step decrease in sediment supply from the end of historic gold mining influence and projections of prolonged drought, it is possible that future sediment supply to San Francisco Bay will decline [45]. We therefore ran four sediment supply scenarios (historic, constant, declining, increase) to explore the potential implications of changing sediment supply to tidal marsh elevations relative to SLR. The historic, constant, and declining scenarios are based upon the projections in Cloern et al. [45]; the historic scenario used the coefficient derived from the soil core calibration and is representative of the sediment available (*C*) over the last century (Table 1), the constant scenario assumed future *C* will only be 60% of historic conditions, and the declining scenario assumed a 1.6% annual decrease in *C* from the constant scenario. The increased sediment supply scenario was conceptually based upon the potential for a wetter future due to climate change, as well as continued land cover changes within the watershed, and assumed a 25% increase in *C* over the historic scenario [46].

#### Organic matter

We explored model projections of elevation and carbon accumulation across several OAR functions. Five single-species models of SOM and a site-specific multi-species community transition model were compared under low and mid SLR scenarios. The site-specific communities were: *S*. *pacifica*, *B*. *maritimus*, and *S*. *foliosa* for Petaluma; *S*. *pacifica*, *S*. *acutus*, and *S*. *americanus* for Rush Ranch; and *S*. *acutus* and *S*. *americanus* for Browns Island. These are based on the most dominant species occurring in our field surveys (S3 Table in S1 File).

#### Salinity

Organic matter production responses to changes in salinity were parameterized based on the results of a greenhouse experiment where tidal marsh plants were grown under a range of salinities [62]. On average there was a 2.7% decrease in total biomass per unit increase in PSU. We ran four scenarios with linear salinity increases of 0, 0.2, 0.4, and 0.6 PSU per decade to simulate salt water intrusion from SLR and changes in freshwater flows [45]. The change in biomass due to salinity was defined as

$$B_{zt}=B_{z}-B_{z}\omega P_{t}$$ (17)

where *B*_*z*_ is the annual organic production for a given elevation, *t* is the time step, *P* is the annual rate of salinity increase, relative to historic conditions, and *ω* is the effect of changing salinity on organic production. For simplicity, this change in productivity was the only effect of elevated salinity that we considered.

#### Sea-level rise threshold

We ran WARMER-2 with linear rates of SLR ranging from 1–10 mm yr^-1^ to estimate a threshold rate for wetland persistence. The model was initiated at the average elevation at each site using average model input parameters and run for 300 years, which was enough time for the model to reach a new equilibrium or drop below the elevation for vegetation establishment. The carbon accumulation rate at the end of the simulation was then compared across sites, productivity functions, and sediment scenarios. The effect of increased salinity was not assessed because the linear nature of the implementation resulted in unrealistic impacts after 300 years.

#### Sea-level rise projections

**Fig 3:**
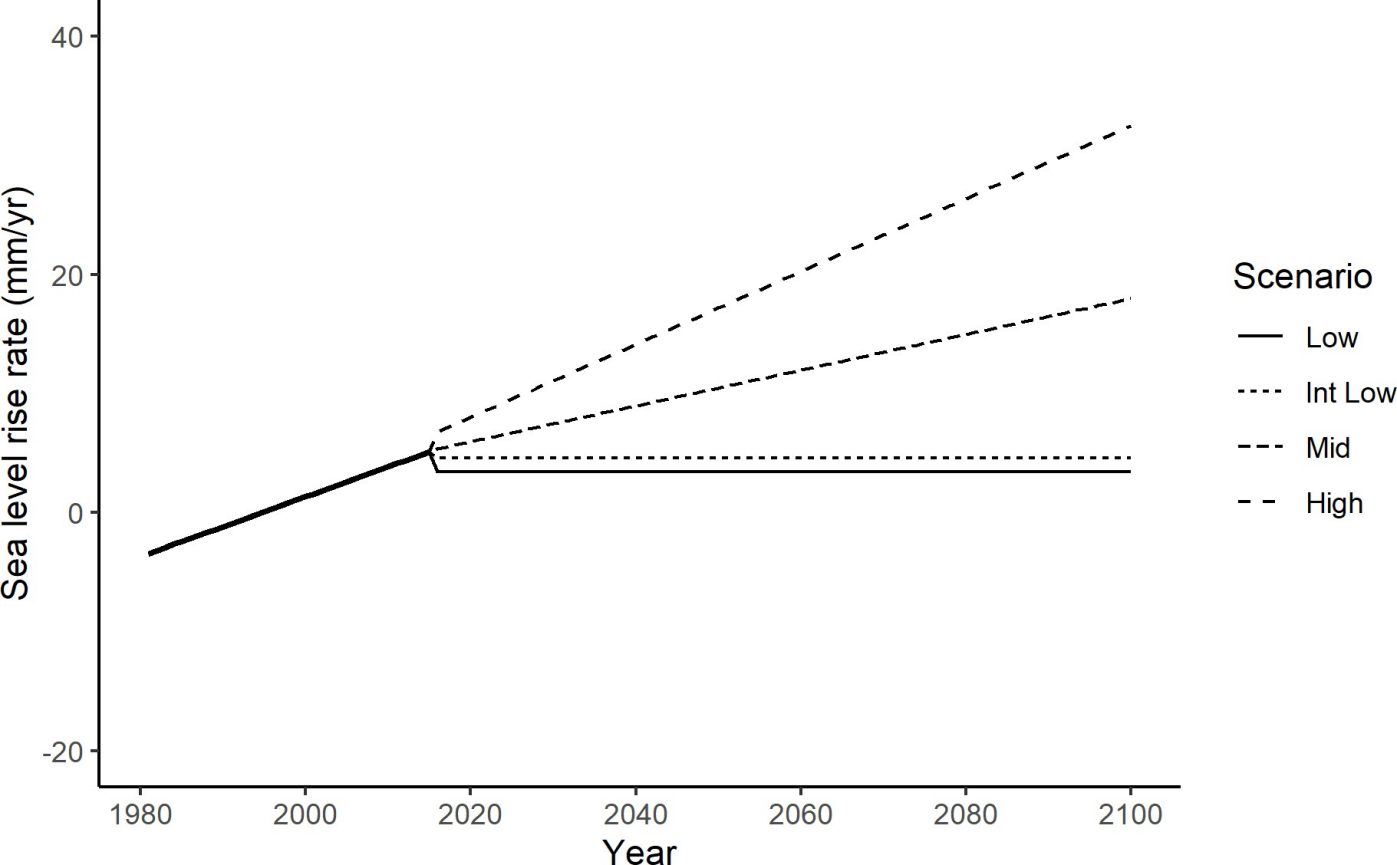
Sea-level rise scenarios. Recent historic and projected rates of sea-level rise for San Francisco Bay. The observed change in annual mean sea level (mm yr^-1^) from 1981–2015 at the Golden Gate gage, relative to 2000 (bold line) and projected rates of sea-level rise from four scenarios [15].

We explored marsh elevation response to four SLR scenarios for the Golden Gate tide gauge at the mouth of San Francisco Bay [15]. The date of the baseline digital elevation model (DEM) (2016) was selected as time zero for all simulations to 2100. We accounted for SLR that has occurred from 2000–2015 (tidesandcurrents.noaa.gov) resulting in low (29 cm), intermediate low (39 cm), mid (99 cm), and high (167 cm) scenarios of total SLR from 2016 to 2100. The low and intermediate low scenarios were linear increases in sea level (3.4 and 4.6 mm yr^-1^ respectively), while the mid and high scenarios were non-linear (Fig 3).

Tide range is also projected to change with SLR, with important implications for wetland processes that are sensitive to inundation. We used results from a hydrodynamic model (Delta Simulation Model 2; DSM2; [68]) for the Sacramento-San Joaquin Delta region to estimate the rate of tide range increase per cm of SLR (cm/cm, Browns Island = 0.061; Rush Ranch = 0.024; DSM2 did not cover the Petaluma River so we assumed an average rate of 0.043). Oscillations in the 18.6-year lunar nodal cycle were also accounted for, with an amplitude of 2 cm. Finally, we incorporated the effects of the El Niño-Southern Oscillation (ENSO) by randomly changing MSL ±5 cm, the average deviation observed during a recent El Niño year [16]; the return interval of ENSO events was set to 4 years.

### Uncertainty

We used 100 Monte Carlo (MC) simulations to incorporate uncertainty in important model parameters. Variation in the mineral and organic matter deposition functions was estimated by first fitting each respective function to the accumulation rates from individual soil cores and then calculating the mean and standard deviation in the fitted coefficient. For each simulation, a coefficient for each accumulation function was randomly selected within one SD of the mean calibration value. Three years of decomposition data for *S*. *pacifica* (Petaluma) and *S*. *acutus* (Rush Ranch and Browns Island) were used to estimate mean (±SD) *A* values for 1, 2, and 3 years [69]. The mean (±SD) influence of salinization on productivity was also selected at random.

## Results

### Validation

The model spin-up SLR rates needed to maintain a constant, average elevation varied with initial elevation (Table 2). Across sites, average spin-up SLR rates for low, mean, and high elevations were 4.74, 3.02, and 2.19 mm/yr, respectively, comparing generally well to the long-term SLR average for San Francisco Bay (2.1 mm/yr). Modeled bulk density and percent organic matter depth profiles aligned closely with the soil core data at Rush Ranch and Petaluma, while at Browns Island the model tended to over-estimate percent organic matter (S7–S9 Figs in S1 File). The WARMER-2 accretion rates also compared well with recent SET measurements, with modeled rates within the standard error of the field measurements at Rush Ranch and Browns Island, but were lower than observed rates at Petaluma (Table 2).

**Table 2:** Model validation results.

	Spin-up sea-level rise (mm yr^-1^)			Accretion rates (mm yr^-1^)	
Site	Mean-SD	Mean	Mean+SD	SET (mean, SE)	WARMER-2
Petaluma	5.81	2.88	2.66	2.64 (0.37)	2.03
Rush Ranch	5.64	2.65	2.68	2.90 (0.53)	2.49
Browns Island	2.78	3.54	1.23	3.32 (2.37)	3.36

Model spin-up sea-level rise rates are shown for mean ±SD elevation of each site.

### Sea-level rise scenarios

**Fig 4:**
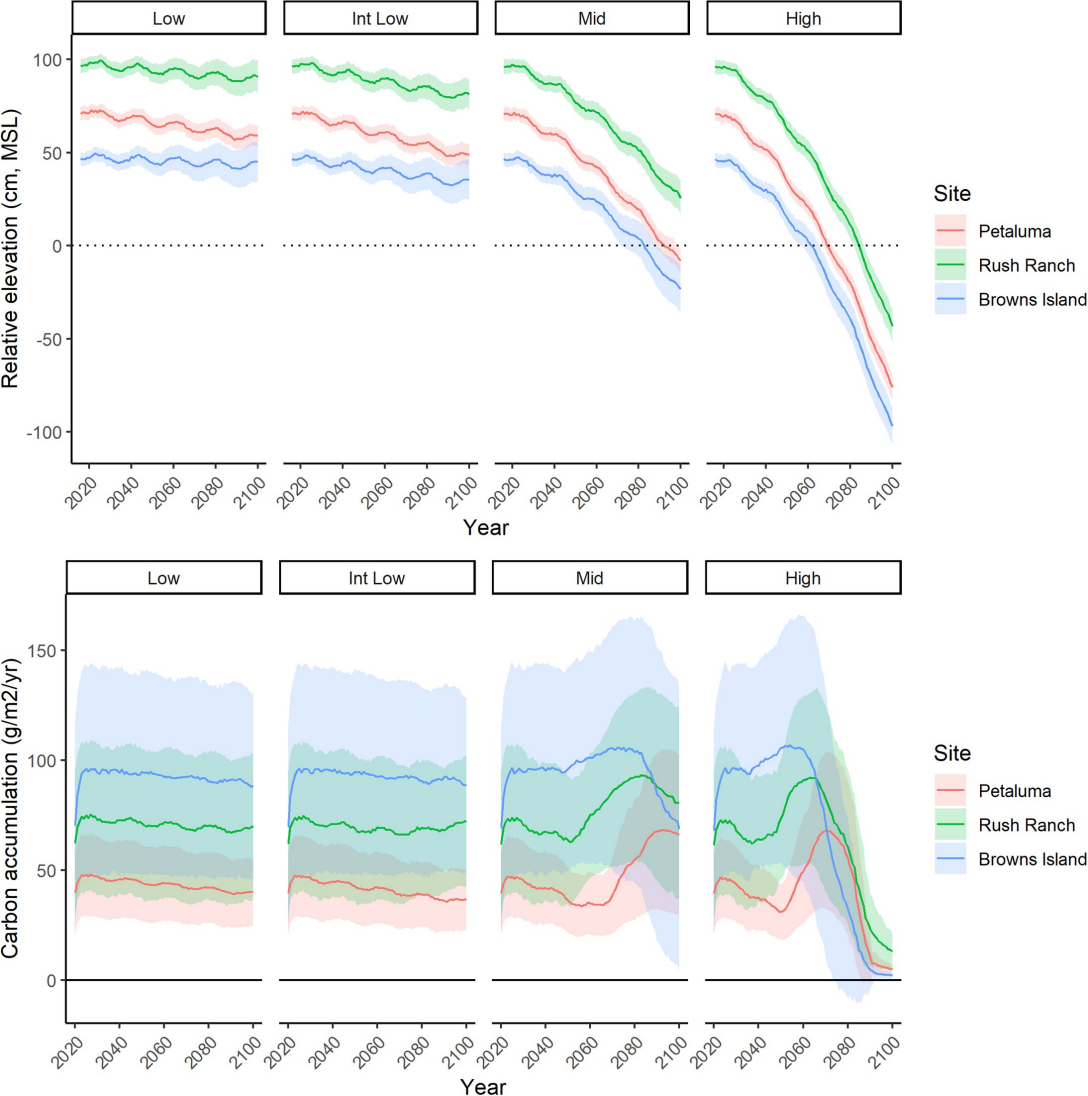
WARMER-2 elevation and carbon projections. Projected mean (±SD) elevation (cm, MSL) and carbon accumulation (g m^-2^ yr^-1^) at three tidal marsh sites across four sea-level rise scenarios (29, 39, 99, and 167 cm by 2100). These projections used the community transition vegetation model, the constant sediment supply scenario, and a 0.2 ppt per decade increase in salinity.

The three sites varied substantially in their initial elevation and potential for upland transgression (Fig 4). Rush Ranch had a relatively high initial mean elevation (0.99 m, MSL, SD = 0.59) due in part to the inclusion of adjacent upland transgression areas in the model domain. Petaluma marsh had a mean elevation of 0.74 m, MSL (SD = 0.37) and included the largest marsh area of our three sites, but it had negligible area available for upland transgression, as we assumed levees would be maintained into the future. Browns Island had the lowest initial mean elevation (0.42 m, MSL, SD = 0.48) and no upland areas available for marsh transgression.

We used the constant sediment supply and the +0.2 ppt per decade salinity scenarios with the community transition productivity function to project changes in wetland elevation, carbon accumulation, and dominant vegetation type through 2100; we felt this combination of scenarios was most the probable and facilitated the evaluation of ecosystem response to SLR. Under the low and intermediate low SLR scenarios, mean marsh elevation declined slightly at Browns Island (mean loss of 8.2 cm), and Rush Ranch (12.1 cm), and declined 15.2 cm at Petaluma marsh (Fig 4). Under the mid SLR scenario, which included an increasing rate of SLR to 2100, mean marsh elevation declined 72–78 cm by 2100 across all sites. The high SLR scenario, with a 167 cm increase by 2100, led to extensive loss in marsh elevation, with an average of 144.3 cm lost relative to MSL across sites. Incorporating uncertainty into model parameter estimates resulted in increasing variation in projected mean marsh elevation and CAR over the course of the simulation (Fig 4).

Estimated mean CAR was inversely correlated with salinity across the estuary. CAR was initially highest at Browns Island, followed by Rush Ranch and Petaluma. Under low and intermediate low SLR scenarios, CAR declined only slightly by 2100. However, under the mid and high SLR scenarios, CAR fluctuated, increasing after 2050 before declining due to transitions of marshes to unvegetated mudflat. CAR at Petaluma showed an increasing trend in the second half of the century, as the dominant species transitioned from *S*. *pacifica* to *S*. *foliosa*. Across all sites and SLR scenarios, there was substantial variation in the projections of CAR due to the range of productivity and decomposition values used in the Monte Carlo simulations.

**Fig 5:**
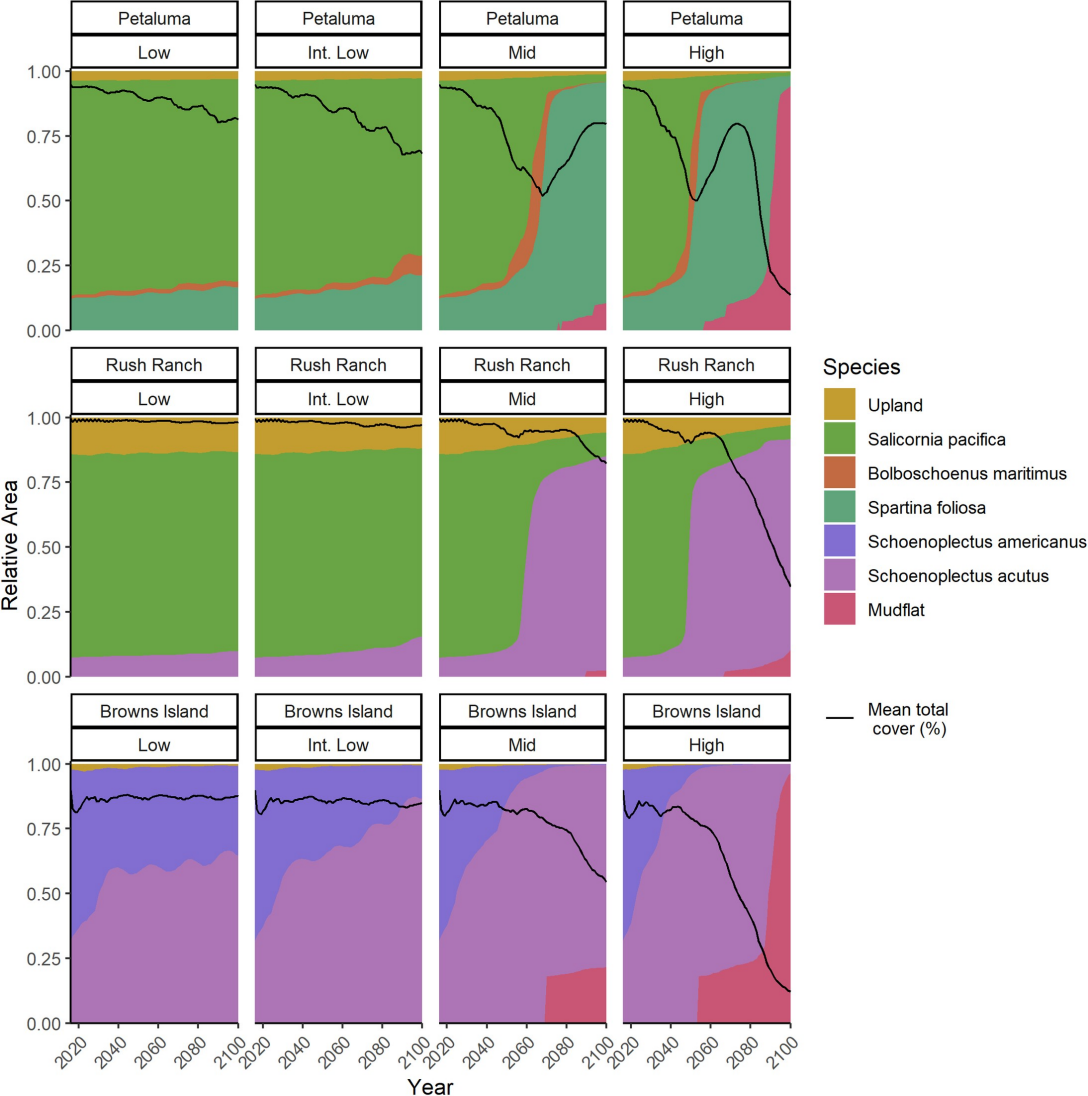
WARMER-2 plant community projections. Projected relative abundance of dominant plant species from the species transition model at three tidal marsh sites across four sea-level rise scenarios (29, 39, 99, and 167 cm by 2100). Mudflat was assigned if total cover of all species was less than 20%. The projections used the constant sediment supply scenario and a 5% per year increase in salinity.

The relative abundance of species in the modeled sites was projected to change with SLR (Fig 5). Initially, Petaluma marsh and Rush Ranch were dominated by *S*. *pacifica*, while two thirds of the vegetation at Browns Island was *S*. *americanus*. While *S*. *americanus* was included in the model for Rush Ranch, it did not reach majority cover under any scenarios, due to its overlap in distribution with *S*. *pacifica* and *S*. *acutus*. Under the two lowest SLR scenarios, WARMER-2 projected only minor changes in species composition at Rush Ranch and Petaluma marsh by 2100, while under the intermediate low scenario Browns Island shifted to dominance by *S*. *acutus*. All sites were projected to be vegetated primarily by flood-tolerant species under mid SLR. Browns Island was projected to lose 25% of its vegetated marsh by 2100. Under high SLR, the conversion to flood tolerant species occurred 15–20 years sooner than under the mid SLR scenario; by 2100, less than 10% of the marsh area was projected to be vegetated marsh at Petaluma marsh and Browns Island. The amount and timing of conversion to unvegetated mudflat was sensitive to the cover threshold we used (20%), although average percent cover across each site declined substantially under high SLR (Fig 5).

### Sea-level rise thresholds

**Fig 6:**
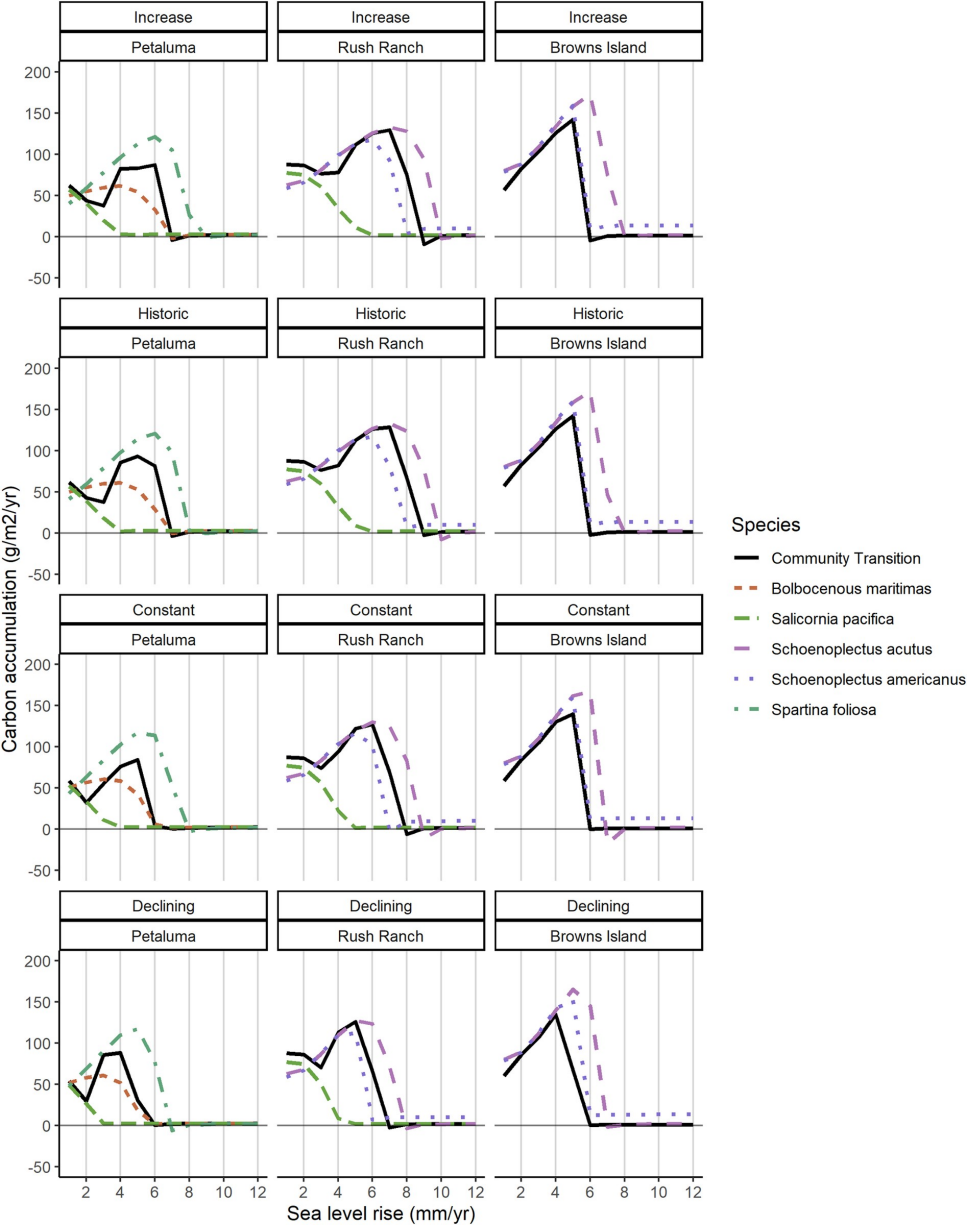
Sea-level rise rate thresholds. Rates of carbon accumulation (g m^-2^ yr^-1^) across a range of linear sea-level rise rates (mm yr^-1^) for each species productivity function and sediment scenario. The model was initiated at the mean elevation for each site and run for 300 years with a constant sea-level rise rate. Carbon accumulation declines to near zero when the marsh does not keep pace with sea-level rise.

Estimated sea-level rise thresholds for marsh persistence ranged from 5–8 mm yr^-1^ and depended on the dominant wetland species contributing organic matter to marsh accretion. Across all combinations of site, sediment, and vegetation, the model projected a mean SLR threshold of 5.6 (SD = 1.8) mm yr^-1^. Except for model runs with the *S*. *pacifica* productivity function, carbon accumulation increased with SLR rate until a threshold was reached and the wetland was no longer at an elevation to sustain vegetation (Fig 6). At Petaluma marsh and Rush Ranch, the community transition productivity function resulted in moderate projections of carbon accumulation compared with the single-species functions, while at Browns Island it resulted in lower carbon accumulation due to the competition between two species with broad niche overlap (Fig 6). The mean SLR rate threshold for marsh persistence was 5.9 (SD = 1.11) mm yr^-1^ across sites and sediment scenarios using the community transition function. Increasing sediment availability raised the SLR rate threshold to 8 mm yr^-1^ at Rush Ranch, while under the declining sediment scenario the SLR rate threshold was only 5 mm yr^-1^ at Petaluma marsh and Browns Island and decreased to 6 mm yr^-1^ at Rush Ranch.

## Discussion

Process-based modeling is an important tool to help inform adaptation strategies that mitigate tidal wetland loss from accelerating SLR and to plan for wetland restoration. Here we updated a modeling approach to assess multiple drivers of ecosystem change (SLR, salinity intrusion, plant community transitions, and sediment availability) on wetland vulnerability to submergence over the coming century, and applied the model to sites along a salinity gradient in the San Francisco Bay estuary. Our results show that under low and moderate SLR scenarios, current wetland extent is likely to be maintained, with only modest loss of relative elevations by 2100. However, wetlands were at greatest risk of losing relative elevation under the highest rates of SLR and under scenarios of reduced sediment availability. These projections broadly align with prior marsh modeling efforts across San Francisco Bay [9, 39, 70] and from other regions along the Pacific coast [31]. Our case study of San Francisco Bay-Delta tidal wetlands shows the sensitivity of different drivers on wetland elevations that are likely to change over the next century under a range of climate change scenarios.

SFBE tidal wetlands are potentially unstable at relative SLR rates above ~7 mm yr^-1^ (Fig 6). While projections of SLR vary widely, most exceed this threshold sometime this century. Without space for upland transgression, extensive loss of wetlands and their associated ecosystem functions is likely to occur, although the exact timing of loss is highly uncertain. For example, at Browns Island under the mid SLR scenario, WARMER-2 projected mean elevation to drop below zero m MSL in 2083 with a one SD range of 2071–2089 (Fig 4). Mean projections from WARMER-2 and the non-linear SLR scenarios (mid and high) show at least some transition to unvegetated mudflat by 2100 (Fig 5).

A data-driven modeling approach is needed when considering how a specific wetland may respond to future conditions. Our projections relied on a combination of field observations and experimental results to constrain various functional responses, define key relationships, and provide empirical distributions for important model parameters. The Monte Carlo simulations produced projections with a measure of uncertainty that increased with simulated year (Fig 4). By accounting for parameter distributions through random interactions in the Monte Carlo simulations, model projections become probabilistic, providing end-users a more transparent result for evaluation. Tidal wetlands are highly variable and complex systems with interacting stress gradients and biogeomorphic feedbacks that control elevation, plant community dynamics, and soil processes. For such systems, a probabilistic model framework should be preferred over a deterministic one, particularly when projections may be used to inform management decisions.

### Local calibration

WARMER-2 was calibrated via site-specific data including radiocarbon dating and other soil core data representing the recent history of each study site. We refined the model calibration procedure to ensure a robust model, including accounting for relative elevation change in the sediment deposition function and decomposition in the calibration of organic deposition. Our approach to mineral calibration, using a moderately complex deposition model that was calibrated to long-term accumulation rates from soil cores, resulted in an estimate of the long-term average SSC over the marsh plain (Table 1). While these estimates are sensitive to settling velocity and water level data, they offer a cross-site metric for comparing sediment availability. The model calibrated well at Petaluma marsh and Rush Ranch (S7 and S8 Figs in S1 File), with good alignment between modeled and observed soil core characteristics. Two of the five soil cores at Browns Island also calibrated well, while organic production was over-predicted in three cores (S9 Fig in S1 File). In this study, we used ^210^Pb dating to determine vertical accretion dates since ^137^ Cs profiles have recently been called into question given the decay rates in wetland soils [71]. In many estuaries dated core information is lacking and should be prioritized to improve modeling efforts to inform management decisions for these ecosystems.

Similar 0-D models of wetland evolution, such as MEM [37], rely on a top-down calibration scheme for site-specific projections; namely the use of SSC and a gross rate of organic production, often parameterized for *S*. *alterniflora*. However, in the San Francisco Bay-Delta estuary *Spartina* is not a dominant genus at many sites, with *S*. *foliosa* usually restricted to lower elevation areas adjacent to marsh channels. *Salicornia pacifica*, the dominant salt marsh species across California, is a particularly difficult species for which to estimate annual production given its succulent growth form [72, 73]. Additionally, the use of channel SSC to inform surface sediment deposition may be problematic given the exponential decline in sediment deposition that occurs with increasing distance from the wetland channel edge [22, 74]; channel SSC may significantly overestimate sediment delivery to the marsh plain.

Petaluma marsh was the only study site modeled in both this study and the original WARMER study [39]. We compared the projections from Swanson et al. [39] with results presented here from the WARMER-2 model run using the same amount of SLR (124 cm, 2000–2100). WARMER-2 projections showed a similar trajectory of elevation through 2050 but were ~17 cm higher in mean elevation by 2100; this difference is most likely due to the community transition model in WARMER-2 that assumes more organic production at lower elevations. Both modeling approaches showed relatively high SLR vulnerability at this site, a surprising result considering the high SSC of the Petaluma River [75]. Swanson et al. [39] questioned whether the low, soil core-derived accretion rates were realistic; however, a recent sediment deposition study [22] found that rates of mineral deposition in the interior of the wetland (737 g m^-2^ yr^-1^) were close to the mean accumulation rates from the soil cores (803 g m^-2^ yr^-1^). Further exploration is warranted, particularly with 2D sediment mass-balance models capable of capturing spatial dynamics and the influence of changing hydrodynamics with SLR [76].

WARMER-2 projections for Rush Ranch and Browns Island (Fig 6) were similar to recent MEM projections at those sites ([9], also calibrated using core data from [53]). WARMER-2 projected a transition to low marsh a few years ahead of MEM. While unique approaches to vegetation modeling and differences in DEMs and sea-level rise curves make a direct comparison of model results difficult, MEM and WARMER-2 produced similar conclusions regarding the vulnerability of each site to SLR.

Core-derived sediment accumulation rates represent a long-term average over decades and may be a better representation of site conditions than shorter-term SSC measurements since the former incorporates stochastic storm events that occur infrequently. Our future projections inherently assume no change in the frequency of stochastic events, which may not be valid since climate change is expected to increase the frequency and intensity of extreme weather [77] that could affect sediment availability from the watersheds or local sediment resuspension. Spatial variation in core-derived accumulation rates can be large, and it is not clear how many soil cores are required to adequately represent a given site. However, by employing a Monte Carlo framework and running a range of sediment supply scenarios, our projections are more likely to bracket the realized future.

### Climate change impacts

Uncertainty on how tidal wetlands will respond to climate change complicates long-term planning and conservation of these important habitats. SLR has been the primary focus of many previous studies, but other climate drivers such as changes in estuary salinity, river flows, tide range, or temperature are addressed less often. Here, we undertook an initial effort to incorporate some of these additional drivers into modeling in the San Francisco Bay-Delta estuary. We found that changes in sediment supply, plant community composition, and salinity had relatively minor effects on wetland elevation through 2100 (S3–S6 Figs in S1 File), with rates of SLR remaining the dominant factor driving elevation change. However, both the salinity scenarios and productivity functions were important in projecting how carbon sequestration, a critical ecosystem function of tidal marshes, may change with SLR. The threshold analysis also revealed a positive relationship between rates of carbon accumulation and long-term SLR, a finding supported by previous empirical and modeling studies [78–81]; this result stems from a combination of increased mineral deposition at lower elevations and higher productivity of low marsh plants.

Both climate change and continued land use change are expected to affect future sediment delivery to tidal wetlands. Sediment supply has been recognized as one of the most important indicators of wetland resilience to accelerating SLR [82, 83]. Our analysis suggested that wetland elevation change is moderately sensitive to a decrease in sediment delivery to the San Francisco Bay-Delta estuary. Lower total precipitation and associated fluvial discharge projected in the future [84] points to a probable reduction in sediment supply to the estuary. However, high fluvial discharge from more intense atmospheric rivers [85] and rain-on-snow weather events [86, 87] could bolster sediment supplies [46], especially as watersheds continue to become urbanized downstream of dams and reservoirs [88]. Sediment may become a more limited resource for wetlands across San Francisco Bay-Delta as tides deliver sediment to new expansive restoration projects [50]; however a bay-wide sediment budget is not yet available.

### Model assumptions and future directions

We made several simplifying assumptions with the WARMER-2 modeling framework. As a 0-D model, WARMER-2 does not consider spatial variability in sediment deposition (such as proximity to tidal channels) nor the evolution of channels or scarp. By calibrating the model with soil cores sampled from the marsh interior, model projections represent the vulnerability of these areas to SLR; areas adjacent to channels are likely to persist longer than WARMER-2 projects. As marshes lose relative elevation and the tidal prism increases, the resulting higher tidal velocities are likely to widen existing channels and re-mobilize sediment for deposition on the marsh plain. While some proportion of sediment may be exported away from marshes, the sediment that remains would help bolster marsh elevation. This would result in greater resilience than we project here, although the scenario of increased sediment supply may account for some of these dynamics.

The species transition model for organic productivity assumes that conditions during relative elevation loss remain favorable for the establishment of species that can tolerate more flooding. To our knowledge, there are not yet examples of SLR induced marsh loss across California estuaries, which are often characterized by mineral deposition and dominance by *S*. *pacifica*, making it difficult to parameterize predictive models. For instance, it is possible that *S*. *pacifica* dominant marshes could collapse before *S*. *foliosa* can establish, resulting in greater vulnerability to sea-level rise than our projections.

The use of an annual time-step in WARMER-2 ignores the potential for feedbacks resulting from extreme storm events. Intertidal areas of estuaries tend to exist as one of two quasi-stable states: intertidal mudflats and tidal wetlands [56, 78] and a strong driver or disturbance may be required to stimulate a state change. A strong atmospheric river storm during an El Niño winter, for example, could interact with higher future sea levels to generate prolonged flooding that causes a shift in the plant community. In the Chesapeake Bay, a microtidal estuary strongly influenced by wind-wave dynamics, upland transgression of tidal marsh is largely controlled by stochastic events [89]. While wind-waves play a smaller role in intertidal habitat evolution in San Francisco Bay due to the larger tidal range, wetlands may be sensitive to stochastic storm events that could alter long-term resilience. By including random ENSO events in the Monte Carlo simulation, we account for some inter-annual variation in sea level, although incorporating the effects of storms was beyond the scope of this effort. However, efforts to incorporate future storms and associated sediment and water level pulses into the WARMER-2 framework are ongoing.

Improving predictions of sediment deposition is important for refining projections of future marsh vulnerability across Pacific coast estuaries, where accretion is dominated by mineral input. At our mineral-dominated study sites, marsh resilience to SLR was tied to sediment scenarios. Sediment deposition is inherently a site-specific process with interacting physical, geomorphic, and biologic factors. While we took a relatively simplistic approach in WARMER-2, there are increasingly sophisticated techniques available to model deposition; however, deciding which model to use depends largely on the research question, as well as data available for calibration. Complex hydrodynamic models, such as Delft3D, ADCIRC, or ROMs, are powerful tools for flood prediction, but they require many parameter decisions and are sensitive to initial and projected boundary conditions. These models are also computationally expensive which generally limits their simulation time and consideration of parameter uncertainty. More simplified models of intertidal evolution that include spatial sediment dynamics are a promising direction [76, 90], especially if they can be linked to models that account for important belowground processes.

Given the number of marsh evolution models available, an ensemble modeling effort could bolster confidence of future projections of tidal wetland vulnerability to SLR. After aligning input parameters, projections would differ based on the underlying assumptions of each model. If completed across a range of wetland ecogeomorphic types, the uncertainty from using a given model could be imputed from the ensemble results. Ultimately, the rate of SLR is likely to be the primary driver of marsh persistence through 2100 and beyond.

## Conclusions

Using a modified version of a tidal marsh SLR response model calibrated with dated soil cores, we found that marshes across the San Francisco Bay estuary are vulnerable to submergence at SLR rates above 7 mm yr^-1^. Rates of carbon accumulation increased with higher SLR rates, and were sensitive to scenarios of salinity intrusion, sediment availability, and plant productivity functions related to inundation. Under the mid non-linear SLR scenario, low marsh was projected to increase in area by 2100, while mudflat area was projected to increase under the high SLR scenario as areas become too low in the tidal frame to support wetland vegetation. When modeling wetland response to SLR, incorporating uncertainty both in the calibration data but also in future climate projections will increase our capabilities in planning for the future. Uncertainty in freshwater flows into estuaries, increasing salinity from saltwater intrusion, and changes in sediment delivery all need to be addressed in models of wetland vulnerability. Assessments of SLR impacts to tidal wetlands need to be placed in broader context of the many geomorphic drivers that can transform an estuary over the coming years. In building a modeling approach that incorporates multiple drivers, we can better understand uncertainty and inform decision makers working to preserve and successfully restore wetlands over the coming decades.

Input dataset and model results are available at Buffington et al (2021; https://doi.org/10.5066/P9G60NJ0).

## Supporting information

S1 File Supplemental information. (DOCX)
