# Supplementary material for: Incorporation of uncertainty to improve projections of tidal wetland elevation and carbon accumulation with sea-level rise
Source: PLoS One. 2021 Oct 20;16(10):e0256707. doi: 10.1371/journal.pone.0256707 (PMC8528310; doi:10.1371/journal.pone.0256707)
Supplement: S1 File — (DOCX) [file pone.0256707.s001.docx]

Incorporation of uncertainty to improve projections of tidal wetland elevation and carbon accumulation with sea-level rise

Kevin J. Buffington, Christopher N. Janousek, Bruce Dugger, John Callaway, Lisa Schile-Beers, Evyan Borgnis Sloane, Karen M. Thorne

Supplemental Information

**Table S1.** Calibrated growth rate parameter for each species

| **Species** | **Growth rate** |
| --- | --- |
| *Bolbocenous maritima* | 0.48 |
| *Salicornia pacifica* | 0.72 |
| *Spartina foliosa* | 0.78 |
| *Schenoplectus americanus* | 0.89 |
| *Schenoplectus acutus* | 0.85 |

**Table S2.** Organic matter calibration coefficients across plant models and study sites

| **Species** | **Petaluma** | **Rush Ranch** | **Browns Island** |
| --- | --- | --- | --- |
| *Bolbocenous maritima*. | 1.6 | NA | NA |
| *Salicornia pacifica* | 1.55 | 1.8 | NA |
| *Spartina foliosa* | 1.6 | NA | NA |
| *Schenoplectus americanu*. | NA | 1.7 | 2.2 |
| *Schenoplectus acutus* | NA | 1.75 | 2.2 |
| Community transition | 1.6 | 1.9 | 2.3 |

**Table S3.** Frequency of occurrence (in plots along vegetation transects) of common tidal marsh species and median elevation at which select common species occurred at the study sites. PET = Petaluma, RRA = Rush Ranch, BRN = Browns Island. SalPac = *Salicornia pacifica*; DisSpi = *Distichlis spicata*; BolMar = *Bolboschoenus maritimus*; SchAme = *Schoenoplectus americanus*; SchAcu = *Schoenoplectus acutus*; SchCal = *Schoenoplectus californicus*.


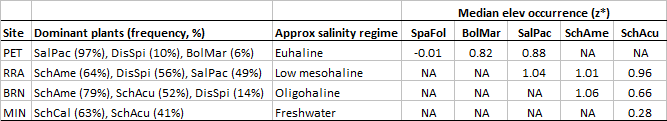


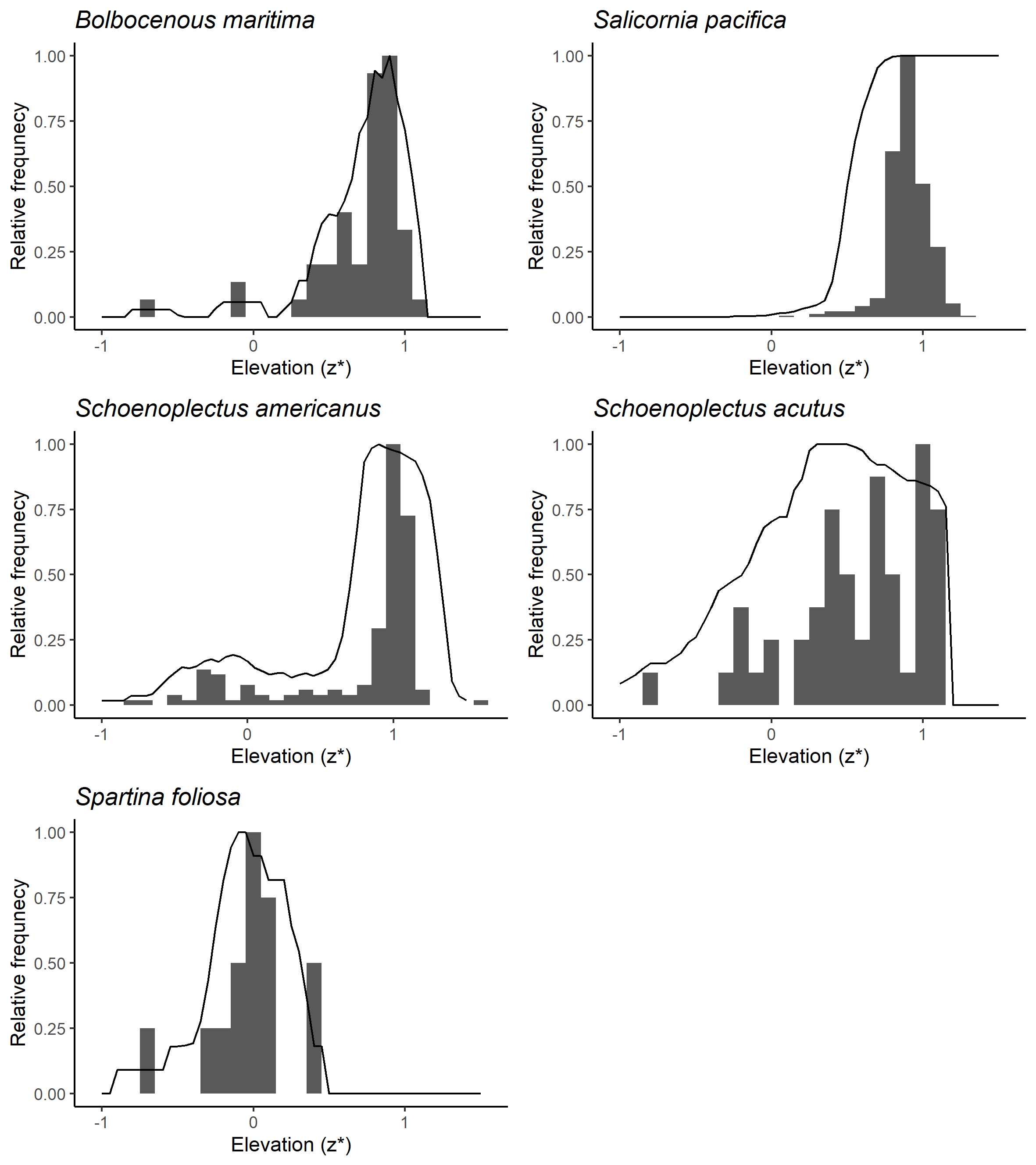


**Fig S1. Species distributions.** Observed relative frequency of species occurrence by elevation in the estuary (bars), and smoothed probability of occurrence functions (lines). z* = (NAVD88-MSL)/(MHHW-MSL)


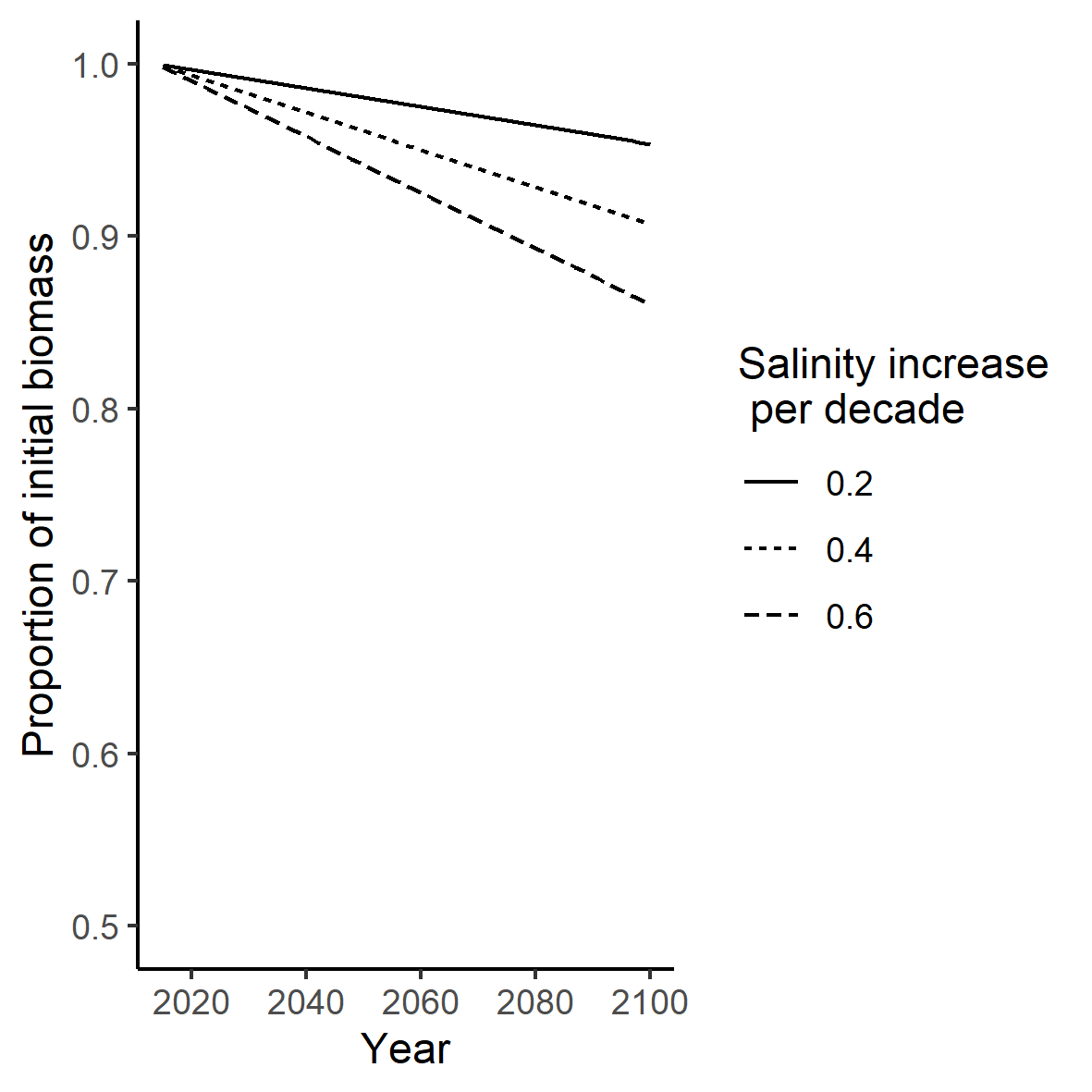


**Fig S2. Salinity scenarios.** Influence of increasing salinity on organic productivity.


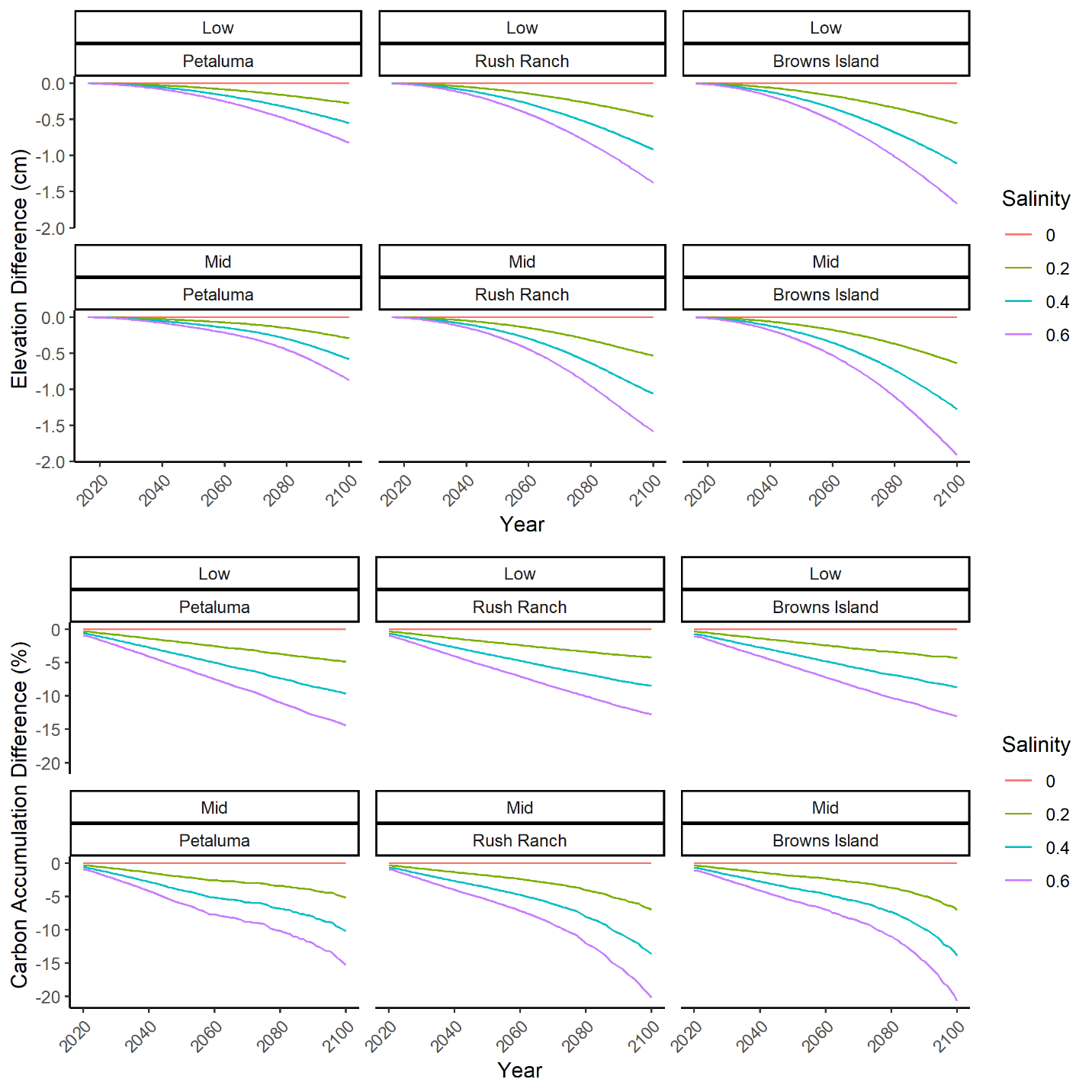


**Fig S3. Senitivity to salinity.** Effects of different rates of salinity intrusion on marsh elevation and carbon accumulation at three tidal marshes across two sea-level rise scenarios (29 and 99 cm by 2100). The different salinity scenarios are a ppt increase in salinity per decade.


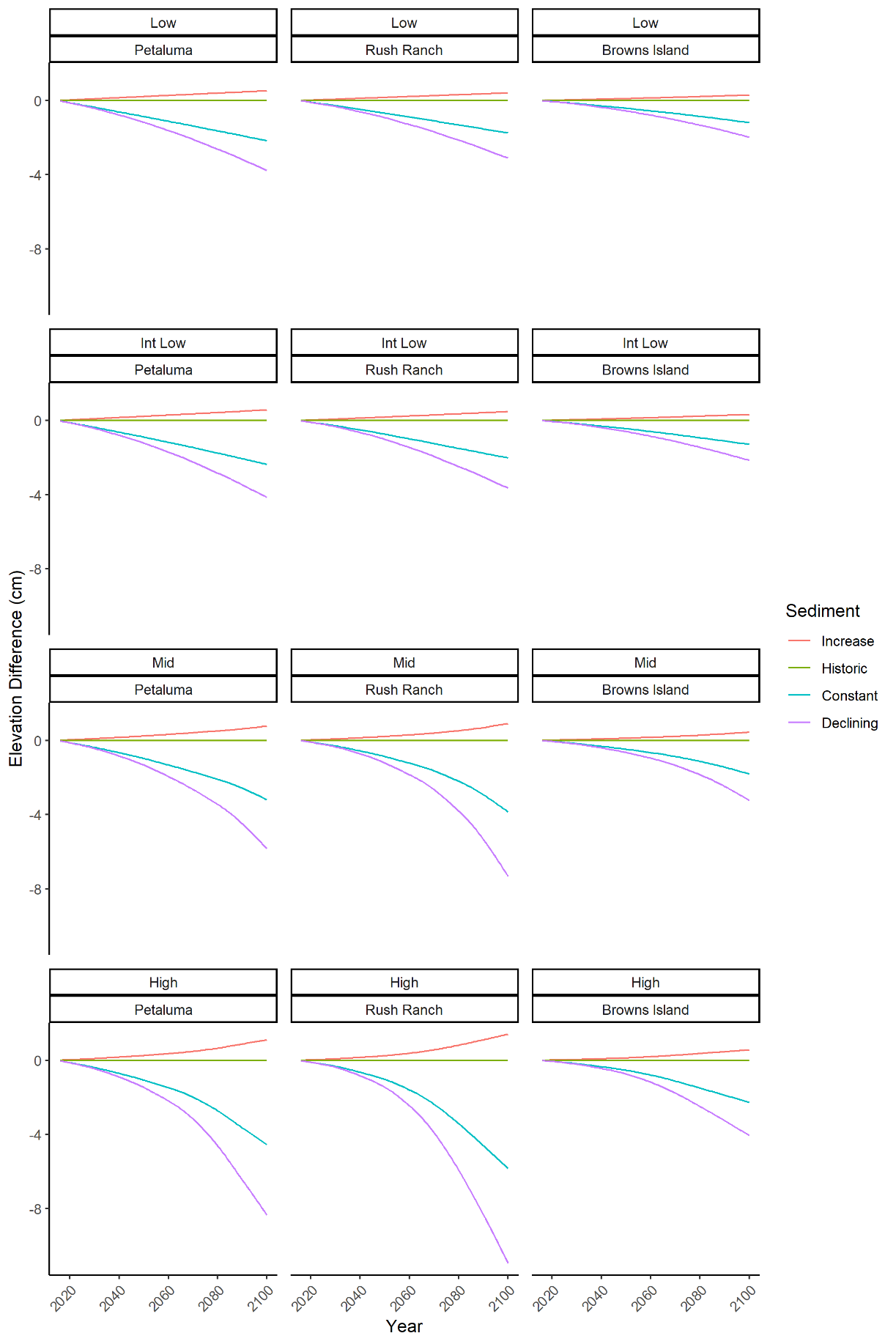


**Fig S4. Elevation sensitivity to sediment supply.** Difference in modeled mean marsh elevation (cm) between historic sediment supply and three other sediment supply scenarios at three tidal marsh sites across four sea-level rise scenarios (29, 39, 99, and 167 cm by 2100).


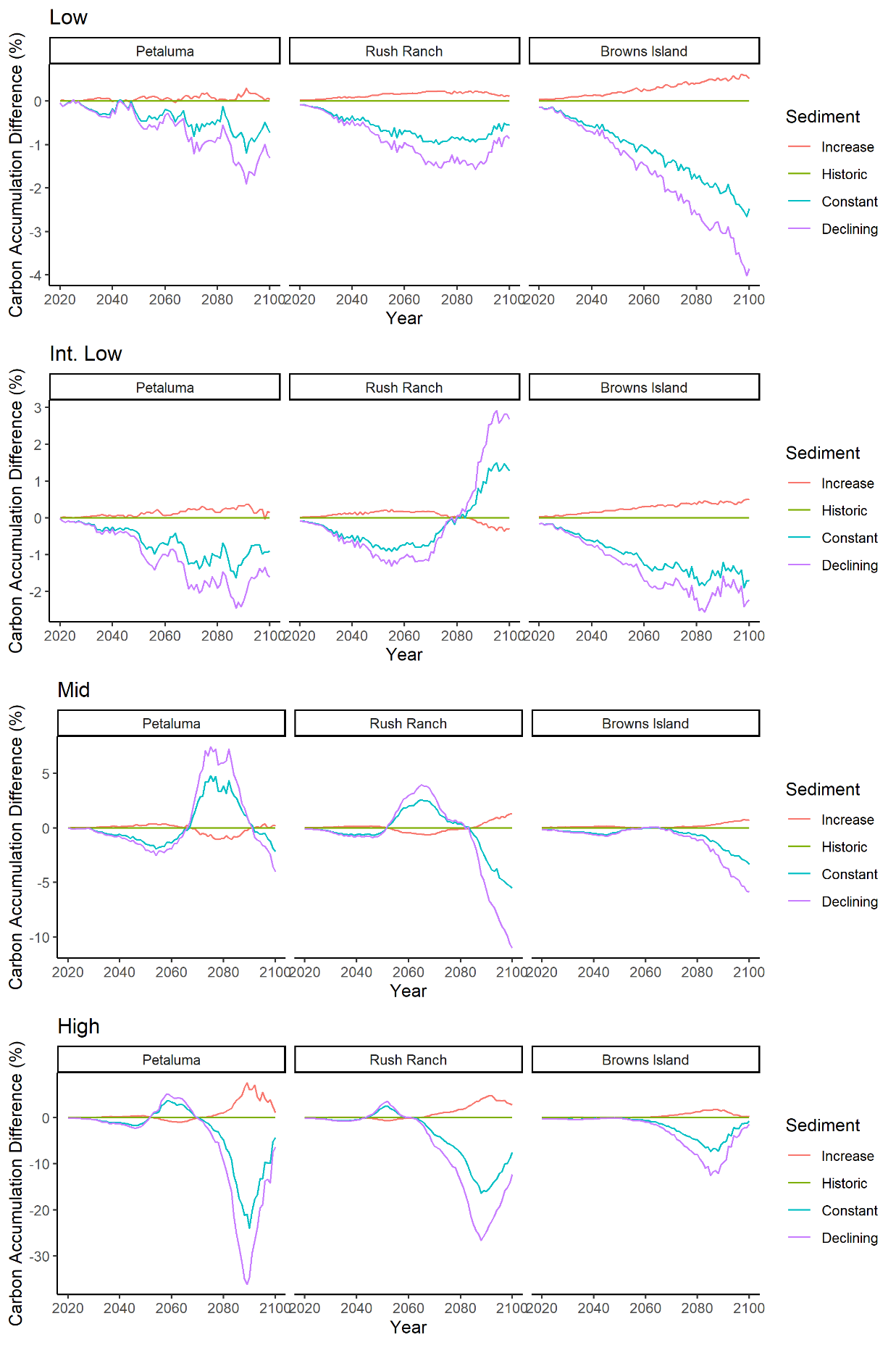


**Fig S5. Carbon sensitivity to sediment supply.** Difference in projected mean carbon accumulation (%) between the historic sediment supply and three other sediment supply scenarios at three tidal marsh sites across four sea-level rise scenarios (29, 39, 99, 167 cm by 2100).


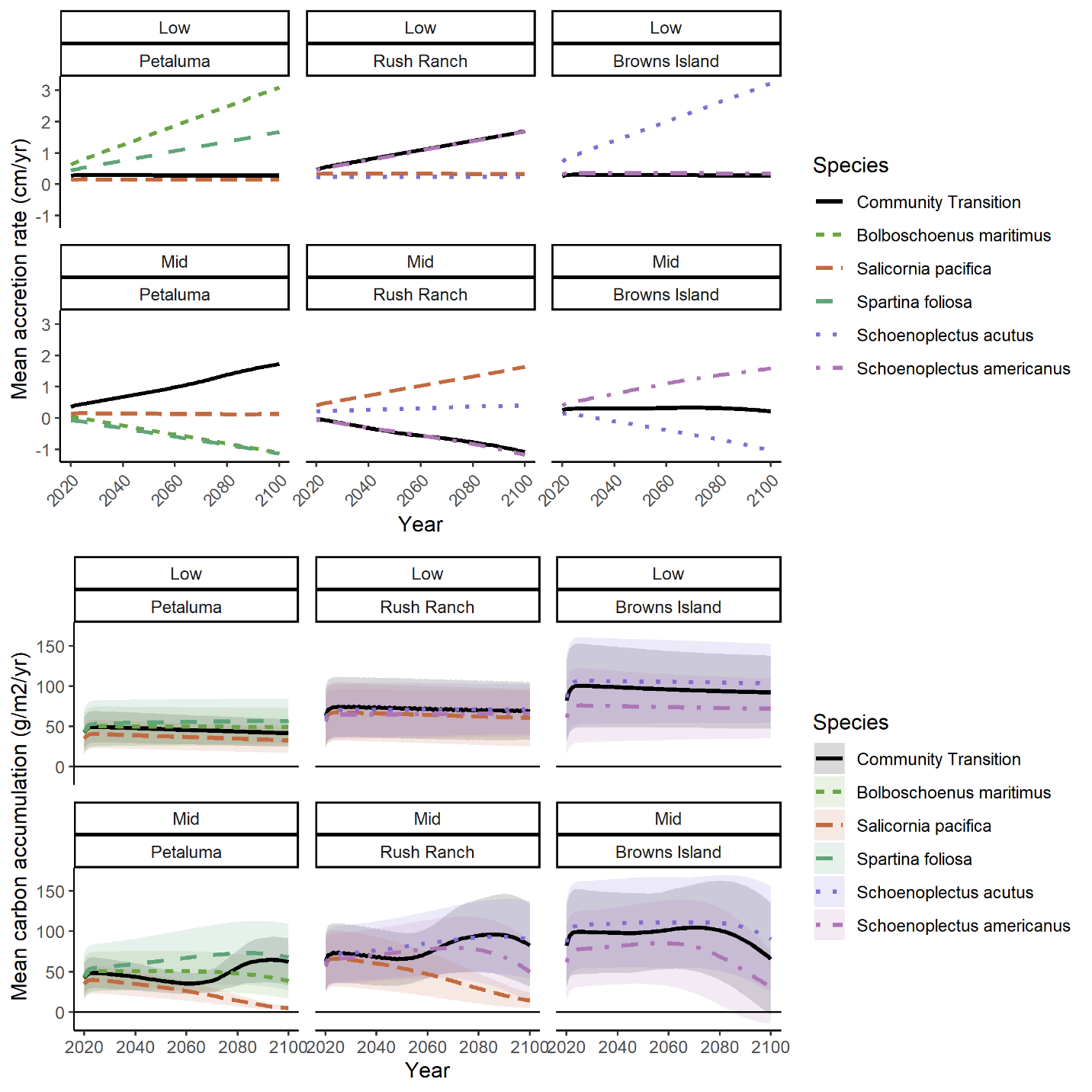


**Fig S6. Sensitivty to organic production.** Differences in accretion rate (cm/yr) and carbon accumulation (SD; g/m2/yr) across different organic productivity functions at three tidal marsh sites across two sea-level rise scenarios (29 or 99 cm by 2100).


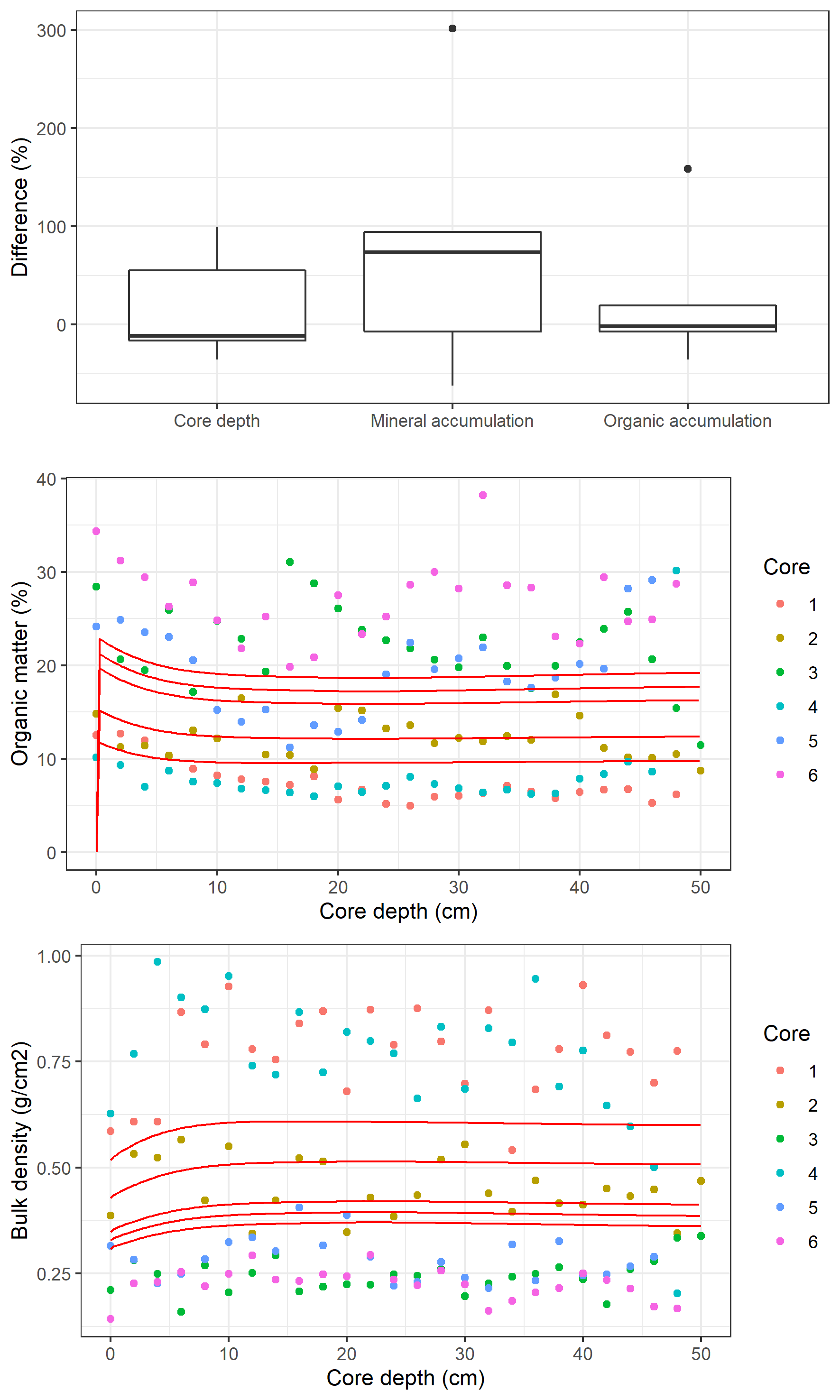


**Fig S7.** **Model calibration at Petaluma marsh.** (A) Difference (%) between observed and modeled soil core depth (accretion rate), and mean mineral and organic accumulation rates. Each soil core was modeled separately for 100 years, using the accretion rate, surface elevation, and amount of sea-level rise to determine the initial elevation. (B) Organic matter (%) with depth for observed (points) and modeled (lines) soil cores. (C) Sediment bulk density (g/cm^3^) with depth for observed (points) and modeled (lines) soil cores.


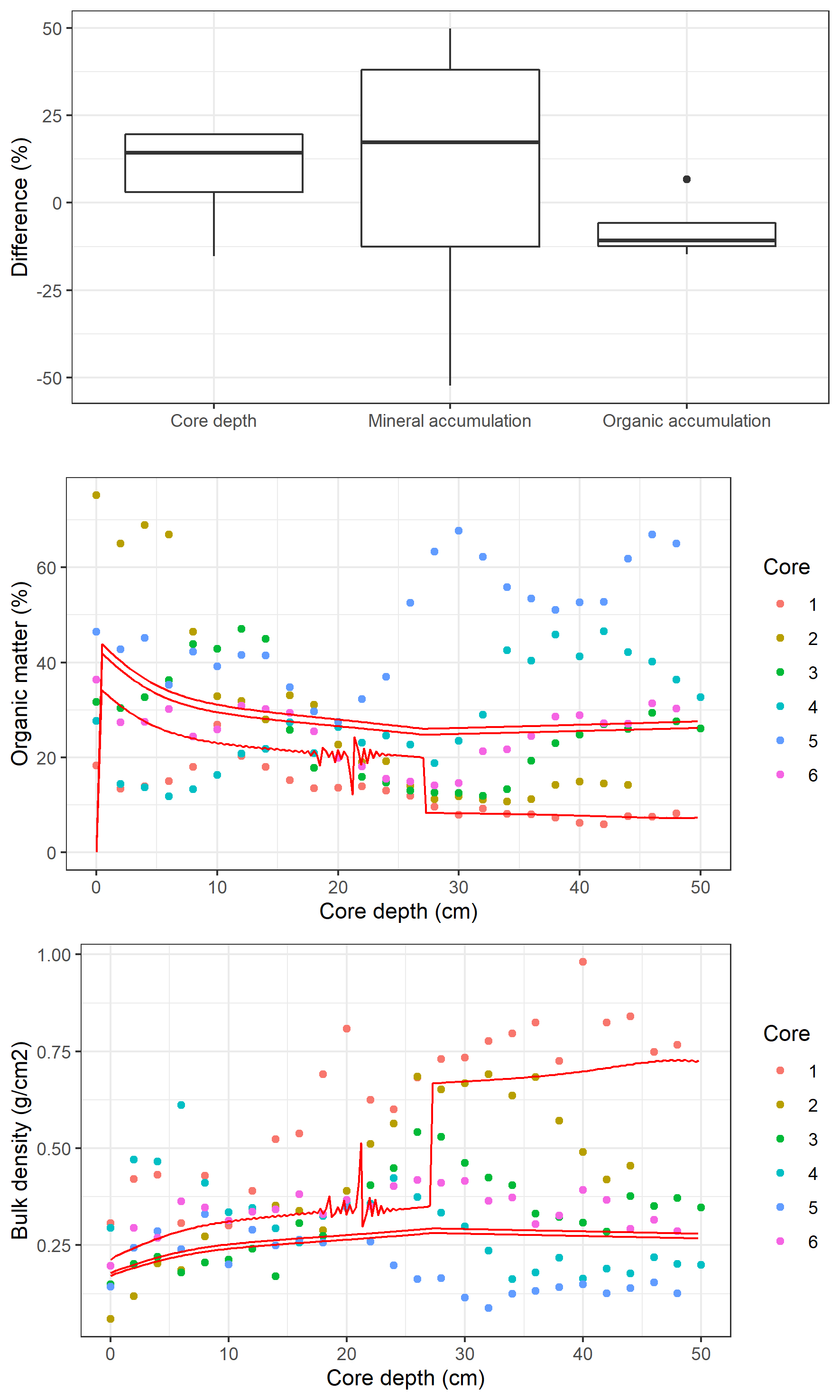


**Fig S8.** **Model calibration at Rush Ranch.** (A) Difference (%) between observed and modeled soil core depth (accretion rate), and mean mineral and organic accumulation rates. Each soil core was modeled separately for 100 years, using the accretion rate, surface elevation, and amount of sea-level rise to determine the initial elevation. (B) Organic matter (%) with depth for observed (points) and modeled (lines) soil cores. (C) Sediment bulk density (g/cm^3^) with depth for observed (points) and modeled (lines) soil cores.


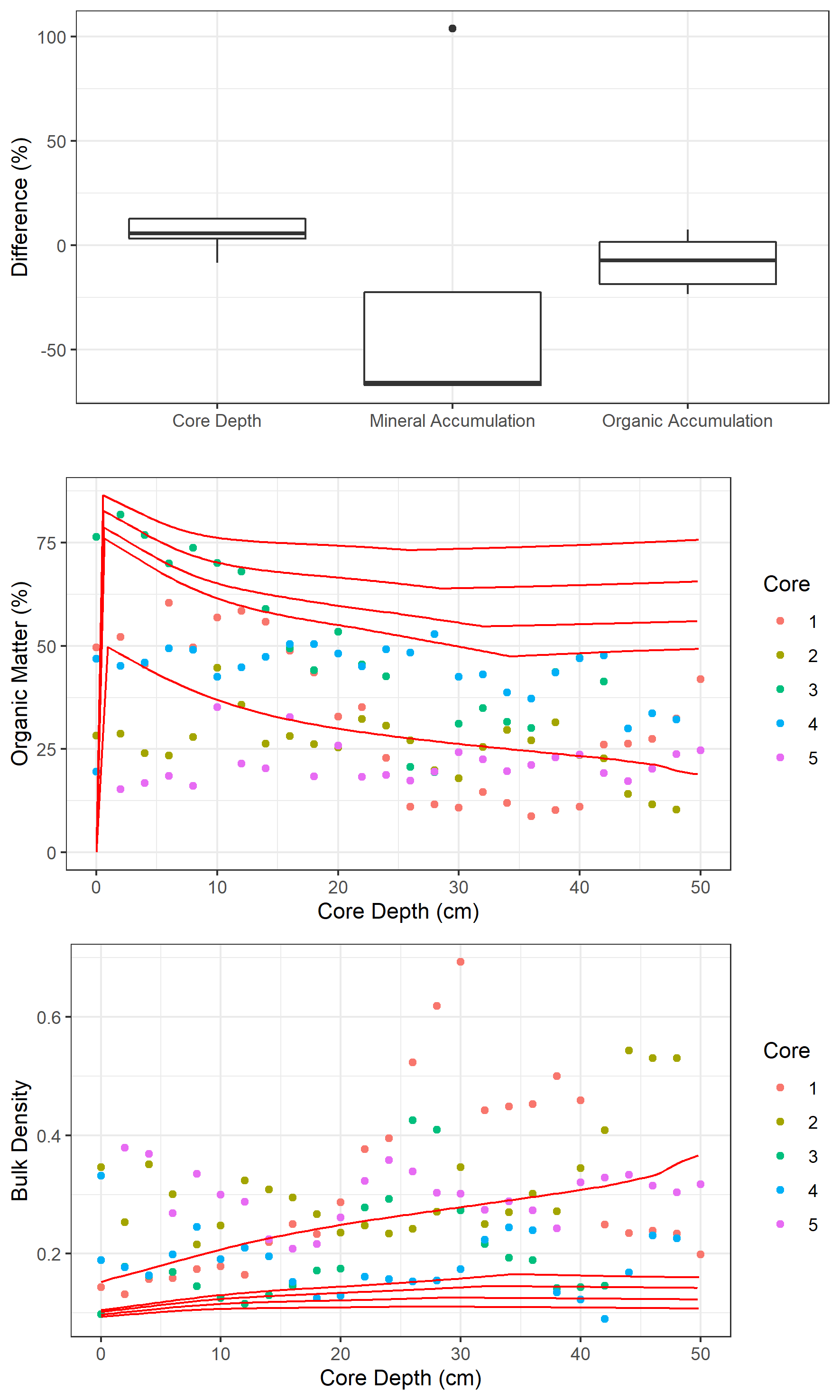


**Fig S9.** **Model calibration at Browns Island.** (A) Difference (%) between observed and modeled soil core depth (accretion rate), and mean mineral and organic accumulation rates. Each soil core was modeled separately for 100 years, using the accretion rate, surface elevation, and amount of sea-level rise to determine the initial elevation. (B) Organic matter (%) with depth for observed (points) and modeled (lines) soil cores. (C) Sediment bulk density (g/cm^3^) with depth for observed (points) and modeled (lines) soil cores.
